# Integration of Repeatome and Cytogenetic Data on Tandem DNAs in a Medicinal Plant *Polemonium caeruleum* L.

**DOI:** 10.3390/ijms26189240

**Published:** 2025-09-22

**Authors:** Olga V. Muravenko, Alexandra V. Amosova, Alexey R. Semenov, Julia V. Kalnyuk, Firdaus M. Khazieva, Irina N. Korotkikh, Irina V. Basalaeva, Ekaterina D. Badaeva, Svyatoslav A. Zoshchuk, Olga Yu. Yurkevich

**Affiliations:** 1 Engelhardt Institute of Molecular Biology, Russian Academy of Sciences, 32 Vavilov St., 119991 Moscow, Russia; 2 All-Russian Institute of Medicinal and Aromatic Plants, Federal Agency for Scientific Organizations, 113628 Moscow, Russia

**Keywords:** *Polemonium caeruleum* L., genome, next-generation sequencing (NGS), repeatome, satellite DNAs, 45S rDNA, 5S rDNA, FISH analysis, chromosome variability

## Abstract

*Polemonium caeruleum* L. (Polemoniaceae) is a perennial flowering plant native to Eurasia and North America, which is used as a fodder, medicinal, and ornamental plant. Many issues related to the taxonomy and origin of this valuable species still remain unclear. The intraspecific genetic variability of *P. caeruleum* and chromosomal organization of its genome are insufficiently studied. For the first time, we analyzed NGS genomic data of *P. caeruleum* using ReapeatExplorer2/TAREAN/DANTE Pipelines. In its repeatome, we identified 66.08% of Class I retrotransposons; 0.57% of Class II transposons; 0.42% of ribosomal DNA; and 0.87% of satellite DNA (six high-confident and three low-confident putative satellite DNAs). FISH chromosome mapping of seven tandem DNAs was carried out in two *P. caeruleum* varieties and two wild populations. Our results demonstrated the effectiveness of using satDNAs Pol_C 46 and Pol_C 33 in combination with 45S rDNA and 5S rDNA for precise chromosome identification. This approach allowed us to study intraspecific chromosomal variability and detect chromosomal rearrangements in the studied accessions of *P. caeruleum*, which could be related to the speciation process. These novel molecular markers are important for chromosome studies within *Polemonium* to clarify its taxonomy and phylogeny, and also, they expand the potential of different breeding programs.

## 1. Introduction

*Polemonium caeruleum* L. (Polemoniaceae), also known as Greek valerian or Jacob’s ladder, is a hardy perennial flowering medicinal plant. This species is widely distributed in Eurasia and North America. *P. caeruleum* plants are 35–120 cm high. They grow mainly in valleys and meadows and along river banks [1,2,3]. *P. caeruleum* is used as a fodder, medicinal, and ornamental plant. This species is also a good honey plant, and it is included in the list of plant species suitable for urban bee-friendly arrangements [4,5]. The main biologically active substances (BAS) of *P. caeruleum* are triterpene saponins [6]. The rhizomes and lower parts of *P. saeruleum* contain up to 30% oleanane derivatives (polmonium saponins, glycosides of theasapogenol derivatives, and β-amyrin), flavonoid glycosides with a predominance of acacetin derivatives, anthocyanins, carotenoid pigments, amino acids, and carboxylic acids [6,7,8,9]. These compounds display a wide range of activity including antioxidant, anti-tumor, anti-inflammatory, and antifungal [8,9,10]. The multifunctionality of the biologically active compounds found in *P. caeruleum* opens up prospects for the development of new effective medicines, and currently, this valuable plant is widely cultivated for the needs of the pharmaceutical industry [11,12].

*P. caeruleum* is a species with a significant range of intra-specific variability in morphological traits and has a high adaptive potential. At the same time, the morphological variability complicates the identification of this taxon and also the differentiation of closely related species within the *P. caeruleum* complex [13,14,15]. In the areas where habitats of the related *Polemonium* species are overlapped, introgressive hybridization events might occur, and several species having a hybrid origin (including, for example, *Polemonium liniflorum* V.N. Vassil. × *P. villosum* Rudolph ex Georgi growing in Siberia) were previously described within this genus [13,16]. Moreover, the subspecies of *P. caeruleum* with overlapped habitats (e.g., subsp. *laxiflorum*, subsp. *yezoense*, and subsp. *campanulatum*), demonstrate both phenotypic similarity and high polymorphism in morphological features [17]. The recent divergence and rapid radiation of the genus *Polemonium,* as well as multiple interspecific hybridization events, may contribute to the emergence of the intra- and inter-specific differentiation of closely related species [2,13,14].

Studies of phylogenetic relationships within the genus *Polemonium* based on AFLP markers as well as nuclear and plastid genome data have clarified some taxonomic issues [15,18]. The intra-specific genetic variability of *P. caeruleum* has been little studied, and the taxonomy and origin of this species also need further clarification [12]

Moreover, there is insufficient information on the structure and chromosomal organization of its genome. The karyotype analysis carried out using the monochrome staining technique indicated the basic chromosome number 2*n* = 2*x* = 18 within the genus *Polemonium* [19,20]. Recently, a FISH-based karyotype analysis of diploid and artificial tetraploid *P. caeruleum* using classical molecular cytogenetic markers, 45S and 5S rDNA, has been carried out. However, in that study, the more accurate identification of several chromosome pairs was needed [12].

Repetitive DNA sequences are a major and fast-evolving portion of plant genomes that can contribute to genome diversity and evolution [21,22]. Tandemly organized repeats are highly dynamic regions which play important roles in genomic variation and gene expression regulation [22,23]. Transposable elements (TEs) and satellite DNA (satDNA) are mostly clustered in heterochromatin-rich chromosomal regions [24,25]. The comprehensive investigation of repeatomes provides a new insight into the organization and evolution of plant species genomes. Currently, molecular cytogenetic markers developed based on the tandem DNA repeats (DNAs) are used for the identification of homologous chromosomes and polyploid subgenomes in cytogenetic studies [26,27,28]. Comparative repeatome studies of related species could clarify their taxonomy and phylogeny [29,30,31].

The breeding of productive and resistant varieties of *P. caeruleum* with a high content of extractive substances requires further study of the genome of this species as well as intra-specific genetic variability [11].

In the present study, for the first time, the repeatome composition in *P. caeruleum* var. ‘Lazur’ was analyzed. FISH mapping of the identified tandem DNAs on chromosomes of *P. caeruleum* was carried out. Based on the chromosome localization of these tandem DNAs, effective molecular markers for precise chromosome identification were determined. Using these markers, the intra-specific chromosomal variability was revealed in karyotypes of two *P. caeruleum* varieties (‘Lazur’and ‘Belosnezhka’) and two wild populations from Russia and Kazakhstan, and structural rearrangements in their karyotypes were detected.

## 2. Results

### 2.1. Identification of DNA Repeats by RepeatExplorer/TAREAN Pipelines

**Figure 1:**
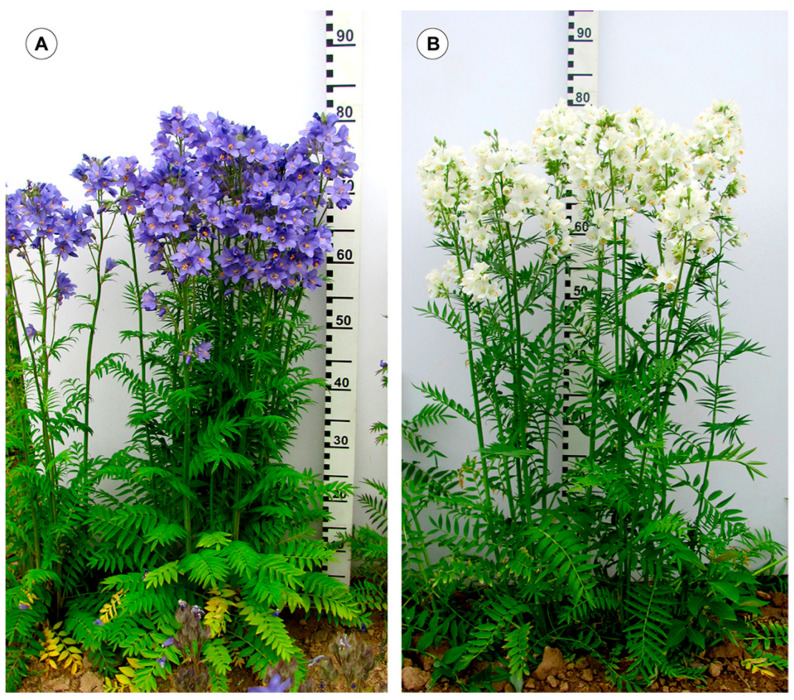
Plants of *Polemonium caeruleum* L. var. ‘Lazur’ (**A**) and var. ‘Belosnezhka’ (**B**).

In this study, we investigated the genomes of plants of *P. caeruleum* var. ‘Lazur’ and var. ‘Belosnezhka’, which were characterized by a high content of extractive substances and differed from each other in the color of their flowers (Figure 1A,B). Moreover, the *P. caeruleum* samples from two geographically distant wild populations (from Russia and Kazakhstan) were also studied.

**Figure 2:**
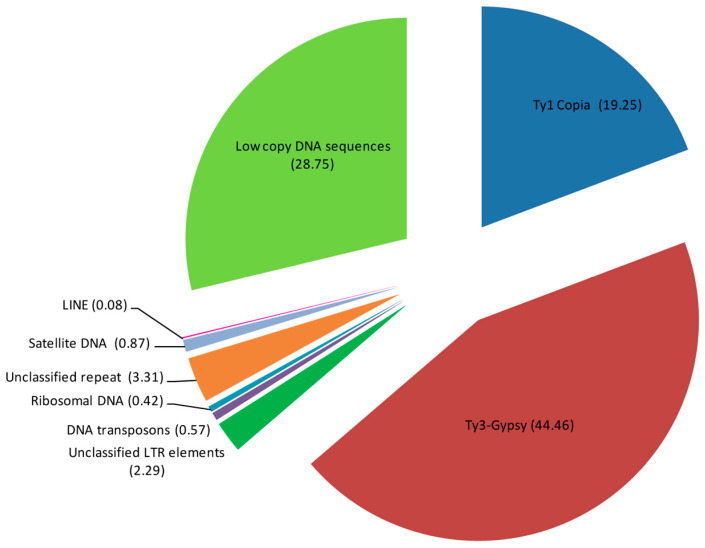
Types and genome proportions (%) of the most abundant DNA repeats identified in the *Polemonium caeruleum* genome. Each proportion was calculated using RepeatExplorer2 as the ratio of the number of reads specific to the particular repeat type to the sum of all reads used in the cluster analysis.

The bioinformatic analysis of the NGS genome data of *P. caeruleum* var. ‘Lazur’ demonstrated that mobile genetic elements constituted the majority of the repetitive DNA (Table 1 and Figure 2). In Table 1, the approximate genome proportions of the most abundant repetitive DNA sequences identified in the genome of *P. caeruleum* var. ‘Lazur’ are represented.

In the *P. caeruleum* repeatome, retrotransposons (Class I) were particularly abundant (66.08%), while DNA transposons (Class II) were present in smaller amounts (0.57%). LTR retrotransposons were the most prevalent mobile elements of Class I, with Ty3-Gypsy elements (44.46%) being more common than Ty1-Copia elements (19.25%). Within the Ty1-Copia superfamily, SIRE (10.7%) and Angela (7.66%) were the most abundant retroelements. In the Ty3-Gypsy superfamily, chromovirus Tekay (32.19%) and non-chromoviral Athila (10.9%) dominated (Table 1). Small proportions of satellite DNA and ribosomal DNA (0.87% and 0.42%, respectively), were detected (Table 1).

Using TAREAN, six high-confiden, Pol_C 33, Pol_C 46, Pol_C 67, Pol_C 70, Pol_C 125, and Pol_C 134, and four low-confidence, Pol_C 1, Pol_C 140, Pol_C 142, and Pol_C 158, putative satellite DNAs were identified in the *P. caeruleum* var. ‘Lazur’ genome (Table S1). At the same time, the DANTE_LTR analysis showed that the tandem DNA repeat Pol_C 1 was a Ty1-Copia SIRE LTR retrotransposon (LTR-RT Pol_C 1). The genome proportion of each tandem DNA repeat and other details including their consensus length are shown in Supplementary Table S1. Among the high-confidence repeats identified in the ‘Lazur’ genome, satDNAs Pol_C 33, Pol_C 46, Pol_C 67, and LTR-RT Pol_C 1, had a higher percentage of their genome proportion (0.12–10%) compared with Pol_C 70, Pol_C 125, and Pol_C 134 (Supplementary Table S1). All these tandem DNA repeats were used in FISH assays to analyze their chromosome distribution and reveal promising potential cytogenetic markers for *P. caeruleum*.

The BLAST (version BLAST+ 2.16.0) analysis of the putative satDNAs did not reveal sequence homology between the identified repeats with the exception of Pol_C 70 and Pol_C 158. These satDNAs were partially (22% and 16%, respectively) overlapped with 81% sequence identity. Within the available NCBI database, sequence homology between the identified satDNAs and the tandem repeats revealed in other species was not detected. At the same time, LTR RT Pol_C 1 showed 70–71% of identity/22–24% of coverage with the *Ananas comosus* var. *bracteatus* genome assembly on chromosomes 2, 5–9, 13, 16–18, 22, and 25. This repeat also demonstrated high sequence homology (70.63% of identity/26% of coverage) with the *Vitis vinifera* retrotransposon V14 (EU009621.1).

We also carried out a comparative repeatome analysis among *P. caeruleum* var. ‘Lazur’ and two wild *P. caeruleum* accessions from Norway (samples ERR5555406 and ERR5555143), which were available in NCBI. According to TAREAN, sample ERR5555406 contained seven high- and three low-confident satDNAs. Sample ERR5555143 contained three high- and nine low-confident satDNAs. Most of the satDNAs identified in the genome of ‘Lazur’ demonstrated high sequence similarity with the satDNAs of both samples (Table 2). However, Pol_C 125 was not found in the genomes of ERR5555406 and ERR5555143.

Moreover, TAREAN did not reveal any LTR retrotransposons in ERR5555406. In sample ERR5555143, only one LTR was found which demonstrated 92% identity with satDNA CL67 from sample ERR5555406 and 92% identity with satDNA Pol_C 33 from ‘Lazur’. Additional DANTE_LTR analysis did not confirm the affiliation of this tandem DNA repeat with LTR retrotransposons. The use of multiple RepeatExplorer2/TAREAN/DANTE Pipelines improves the confidence in repeat identification.

### 2.2. Chromosomal Localization of Tandem DNAs

**Figure 3:**
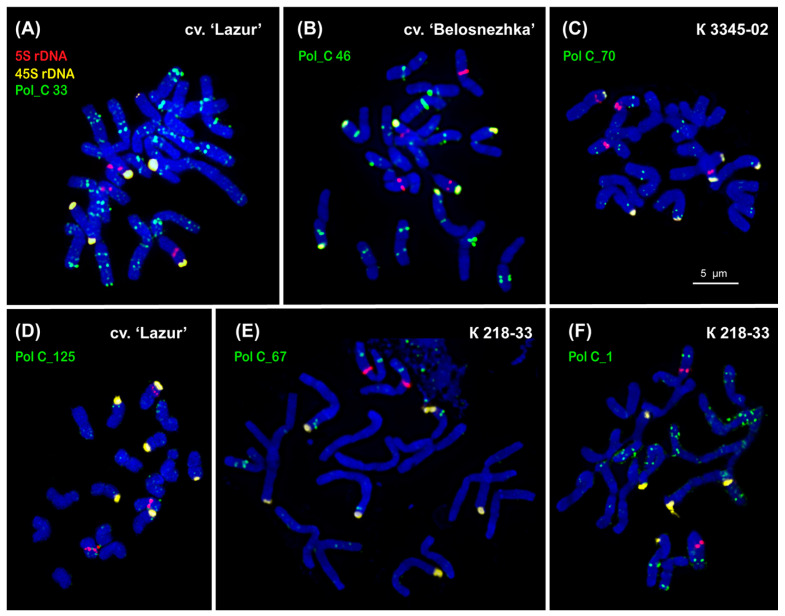
FISH-based chromosome localization of 5S rDNA (red), 45S rDNA (yellow), and tandem DNA repeats (green), (**A**) Pol_C 33, (**B**) Pol_C 46, (**C**) Pol_C 67, (**D**) Pol_C 70, (**E**) Pol_C 125, and (**F**) LTR-RT Pol_C 1, in the studied accessions of *Polemonium caeruleum*. Correspondent probes and their pseudocolours are specified next to the metaphase spreads. Bar—5 μm.

**Figure 4:**
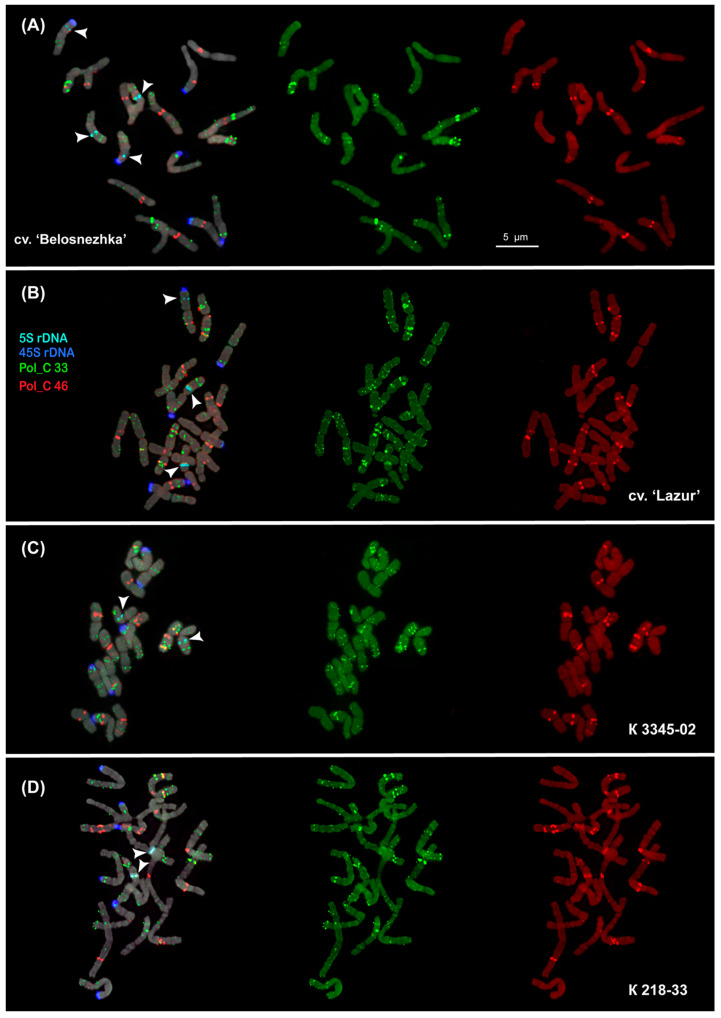
(**A**–**D**) FISH-based localization of 5S rDNA (aqua), 45S rDNA (blue), Pol_C 33 (green), and Pol_C 46 (red) in the metaphase spreads of the studied accessions of *Polemonium caeruleum*. The correspondent probes and their pseudocolours are specified on the left. Heads of the arrows point to the sites of 5S rDNA. Bar—5 μm.

The karyotypes of the studied *P. caeruleum* specimens had 2*n* = 18 chromosomes (about 4–6 µm in length). We carried out a FISH-based chromosome mapping of the seven identified tandem DNA repeats (Pol_C 33, Pol_C 46, Pol_C 67, Pol_C 70, Pol_C 125, Pol_C 134, and LTR-RT Pol_C 1) and also classical chromosomal molecular markers (45S rDNA and 5S rDNA) in *P. caeruleum* var. ‘Belosnezhka’ and var. ‘Lazur’ as well as two wild specimens from Russia and Kazakhstan (Figure 3 and Figure 4).

**Figure 5:**
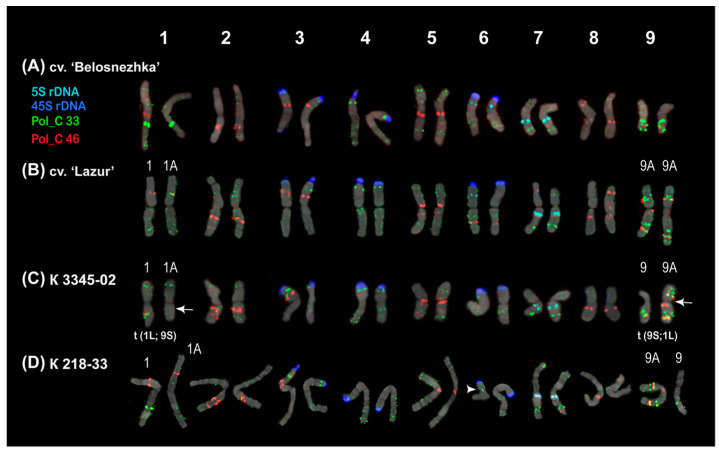
(**A**–**D**) Karyotypes of the studied *Polemonium caeruleum* accessions after FISH with 5S rDNA (aqua), 45S rDNA (blue), Pol_C 33 (green), and Pol_C 46 (red) (the same metaphase plates as in Figure 4). Arrows point to the translocation t (1; 9). The head of an arrow points to the rearranged version of chromosome 6 with a putative deletion.

The major 45S rDNA clusters were observed on the short arms of the satellite chromosome pairs 3, 4, and 6. The major 5S rDNA clusters were localized in the proximal regions of the long arms of chromosome pair 7, and also minor 5S rDNA polymorphic loci were detected in the short arm of chromosome pair 6 (Figure 4 and Figure 5).

The clustered and/or dispersed chromosome distribution of the studied DNA tandem repeats was revealed. Clusters of Pol_C 67 and Pol_C 70 Pol were revealed on some chromosomes where they were usually colocalized with Pol_C 33 (Figure 3). Pol_C 125 and Pol_C 134 were distributed dispersedly along the chromosomes (Figure 3). LTR-RT Pol_C 1 was colocalized with Pol_C 33 on some chromosomes (Figure 3). The localization of Pol_C 33 and Pol_C 46 clusters presented chromosome-specific distribution patterns in the *P. caeruleum* karyotype (Figure 3 and Figure 4).

Clusters of Pol_C 33 were distributed in the intercalary and subtelomeric regions of all chromosomes, and some of them were polymorphic. On the satellite chromosome pairs 3 and 6, clusters of Pol_C 33 and 45S rDNA were colocalized (Figure 3, Figure 4 and Figure 5).

Pol_C 46 clusters were revealed in the intercalary and pericentromeric regions of chromosomes except for chromosome pair 4. On chromosome pairs 1, 7, and 9, some Pol_C 46 and Pol_C 33 clusters were colocalized and they differed in size (Figure 3, Figure 4 and Figure 5).

**Figure 6:**
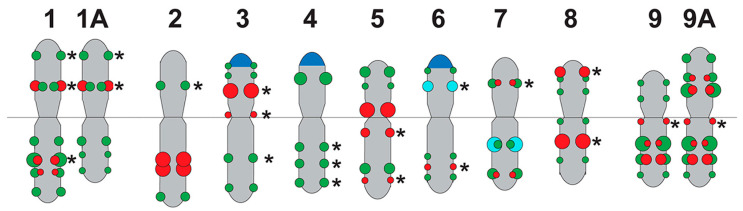
FISH-based idiogram-scheme representing the localization of clusters of 45S rDNA (blue), 5S rDNA (aqua), Pol_C 33 (green), and Pol_C 46 (red) on chromosomes of *Polemonium caeruleum*. Chromosome numbers and different variants of chromosomes 1 and 9 (chromosomes 1 and 1A, and also 9 and 9A) are indicated at the top. Asterisks indicate the polymorphic sites.

The chromosomal distribution of molecular markers Pol_C 46 and Pol_C 33, in combination with 45S rDNA and 5S rDNA, demonstrated an individual pattern for each of the nine chromosomes in the *P. caeruleum* karyotype, which made it possible to identify all homologous chromosome pairs in the karyotype of this species (Figure 4 and Figure 5). As a result, an idiogram-scheme showing the localization of these molecular markers on chromosomes of *P. caeruleum* was constructed (Figure 6).

In the karyotype of *P. caeruleum* var. ‘Lazur’, a balanced variant of chromosomes 1A and 9A (1A and 9A) was revealed, which resulted from a reciprocal translocation t (1; 9) (Figure 5C and Figure 6). In karyotypes of plants from wild populations (K 3345-02 and K 218-33), different variants of chromosomes 1 and 9 (chromosomes 1, 1A, and also 9, 9A) were observed. Moreover, another chromosome rearrangement (chromosome 6 with a putative deletion) was detected (Figure 5).

## 3. Discussion

Plant genomes include a large number of repetitive DNA sequences [32,33,34]. Transposable elements (TEs) constitute up to 90% of their genomes [33,34,35]. The genome size expansion or reduction is lineage-specific in the plant taxonomy. In plant genomes, fractions of mobile elements might also vary, ranging from ~3% in small genomes to ~85% in large genomes, which indicate a linear relationship between genome size and mobile element content [33]. In particular, genome size correlates with the prevalence of LTR retrotransposons [36,37,38].

LTR retrotransposons are the most abundant repeats in the *P. caeruleum* genome (66.08%). The number of Ty3-Gypsy elements is approximately twice as large as that of Ty1-Copia elements. The same ratio of Ty3-Gypsy/Ty1-Copia proportions was also reported earlier for other plants including *Hydrangea* sp., *Arachis* sp., and *Salvia* sp. [29,31,39,40]. Among plant taxa, individual lineages of Ty1-Copia had narrower distribution than Ty3-Gypsy. The Ty1-copia superfamilies were reported to be more evolutionarily scattered and smaller in size than the Ty3-Gypsy [30,36]. In the present study, the revealed homology of LTR RT Pol_C 1 with genome sequences of other plant taxa (*Ananas comosus* and *Vitis vinifera*) could be related to the significant contribution of Ty1-Copia retrotransposons to plant genome organization and evolution [30,36,38].

LTR retrotransposons might form tandem arrays. When a considerable genome fraction consists of mobile elements, new insertions (even randomly occurred) often arise within or near another mobile element [41]. These structures are highly polymorphic and often found in plant genomes having different size, complexity, and ploidy levels [42]. In addition to autonomous elements, plant genomes might include defective mobile elements, which are difficult to identify due to the ever-increasing accumulation of mutations [43]. The interactions between satDNA and mobile elements may promote the formation of new sequences which might integrate and/or combine structural components of satDNAs and TEs [44,45].

As reported earlier, in the genome of *Pennisetum purpureum* Schumach., Ty1-Copia sequences with high copies were localized in the centromeric and distal regions of chromosomes [46]. Ty1-Copia mobile elements were also identified in the terminal, interstitial, and centromeric/pericentromeric regions of chromosomes in *Coffea eugenioides* S. Moore [47]. On chromosomes of *P. caeruleum*, clusters of LTR-RT Pol_C 1 and satDNA Pol_C 33 were colocalized and mainly distributed in the DAPI-positive heterochromatic regions of chromosomes.

SatDNAs are known to evolve more rapidly than other genome sequences. These repeats often exhibit high polymorphism in array length since they vary in copy number and nucleotide composition even among related species and generations [22,23]. Such rapid changes are thought to drive genomic reorganization [21]. Conversely, some satDNA sequences are remarkably conserved across long evolutionary periods, probably due to their interaction with heterochromatin-associated proteins, which contributes to their potential regulatory role in gene expression [22,23,48]. SatDNAs also exhibit high diversity both within and between populations including multiple variations in monomer sequence, copy number, and chromosome distribution, which may contribute to genome plasticity and population divergence [22,49]. In this study, we revealed the differences in the number of high- and low-confident satDNAs (7/3 vs. 3/9) between the sequencing data of two Norwegian *P. caeruleum* samples which were taken from the database. These differences could be related to both biological and/or technical variations. At the same time, the genuine polymorphism in FISH-based satDNA chromosome patterns observed within and between *P. caeruleum* populations could indicate the adaptation and speciation processes that occurred in this species.

Within a plant genus, satDNAs usually vary among related species in their genomic abundance and chromosomal distribution patterns [50]. The evolution of species-specific satDNAs may result from changes in the copy number of the satellite sequences shared by a group of species, which might be explained by an increase or decrease in the quantity of the repeat copies within the genome. Moreover, DNA repeats can undergo cycles of expansion, contraction, and/or reorganization [51,52].

In the present study, the repeatomes of three studied specimens of *P. caeruleum* contained five common homologous repeats (93–100% of identity) with almost the same length and genome proportions although intra-specific variations in their length and degree of homology were also revealed. Three of them had high genome proportions in the studied accessions of *P. caeruleum*.

Depending on the plant species, tandem DNA repeats can be dispersed along the chromosomes or distributed in clusters in different chromosome regions [29,39,47]. In *P. caeruleum*, we also observed dispersed and/or clustered chromosome distribution patterns of the studied tandem DNAs. Small clusters of the repeats were mostly localized in the pericentromeric and subtelomeric regions of chromosomes, and large clusters were revealed in the intercalary regions. The similar distribution pattern of tandem DNAs was previously observed in many cereals having large chromosome sizes, for example, in *Aegilops* and *Deschampsya* species [27,53].

In karyotypes of the studied specimens of *P. caeruleum*, the Pol_C 33 cluster was detected in the NOR (nucleolar organizer region) in colocalization with 45S rDNA signals, suggesting that some satDNAs (e.g., Pol_C 33) could be distributed across these rDNA arrays. The repeats Pol_C 70, Pol_C 125, and Pol_C 134, having the lowest proportion in the genome, showed very weak FISH signals in the intercalary regions of chromosomes, and its distribution is very similar and partially coincides with the localization of Pol_C 33. A similar satDNA distribution pattern is observed in the genomes of such plants as *Beta* sp., *Hedysarum* sp., and *Hydrangea* sp. [28,39,54].

The similarity in the size and morphology of most chromosomes observed in the karyotype of *P. caeruleum,* as well as weak DAPI banding patterns, make the accurate identification of chromosome homologues difficult. The use of classical molecular chromosome markers, such as 45S and 5S r DNA, contributed to more accurate chromosome identification in the karyotype of *P caeruleum* [12]. In the present study, the analysis of the chromosomal localization of the identified tandem DNAs showed that the distribution patterns of the two most common satDNAs, Pol_C 33 and Pol_C 46, are chromosome-specific and can be used as molecular markers for the identification of chromosome pairs in the *P. caeruleum* karyotype. A combination of four molecular markers, 45S and 5S rDNA, Pol_C 33, and Pol_C 46, provided a unique distribution pattern for each of the nine *P. caeruleum* chromosome pairs, which allowed us to represent a complete and accurate identification of chromosome pairs in the karyotypes of this species. Based on the analysis of chromosome distribution patterns of these molecular markers in the studied *P. caeruleum* specimens, several chromosomal translocations were revealed.

SatDNA repeats represent recombination “hotspots” which contribute to genome reorganization. It has been shown that satDNAs can induce chromosomal rearrangements that directly affect the evolution of the karyotype [55,56]. Chromosomal rearrangements are an important source of genetic variability that influences gene expression in plant genomes with a wide range of phenotypic and metabolic consequences [57].

Polyploidy and chromosome rearrangements are considered to be speciation-related events which are factors influencing the evolution of plant genomes [58,59]. *P. caeruleum* is thought to belong to an evolutionarily young genus [15,60], and the chromosomal rearrangements detected in the karyotypes of different populations of this species could also be a speciation-related process. Moreover, cytogenetic abnormalities including chromosome rearrangements could appear in a plant population under the influence of various environmental stress factors [61,62]. It cannot be ruled out that certain variants of rearranged chromosomes may persist in geographically distant populations of *P. caeruleum*.

Thus, our findings indicate the effectiveness of using four molecular markers, 45S rDNA, 5S rDNA, Pol_C 33, and Pol_C 46, to identify homologous chromosome pairs in the *P. caeruleum* karyotypes. For the first time, a FISH-based idiogram-scheme of *P. caeruleum* chromosomes was constructed. Moreover, chromosomal rearrangements within a systematically complex species, *P. caeruleum,* were detected. The presence of the chromosomal rearrangements in karyotypes of the studied specimens from geographically different populations is consistent with high intraspecific polymorphism in morphological features observed earlier in this perennial species [1,2,3,13]. This approach allowed us to study intra-specific chromosomal variability and detect chromosomal rearrangements in the studied accessions of *P. caeruleum*, which could be related to the speciation process. These novel molecular markers can be used in chromosome studies within the genus *Polemonium* to clarify its taxonomy and phylogeny, and also, they expand the potential of different breeding programs.

## 4. Materials and Methods

### 4.1. Plant Material

The seeds of four *P. caeruleum* accessions were obtained from the collection of the All-Russian Institute of Medicinal and Aromatic Plants, Moscow, Russia. Among them, the accessions of *P. caeruleum* var. ‘Lazur’ (K 26-3287) and *P. caeruleum* var. ‘Belosnezhka’ (K 74-24) were developed and cultivated in the trial plots of the AIMAP Botanic Garden. The *P. caeruleum* plants of accession K 218-33 grew in the natural habit on the right bank of the Ili River in southeast Kazakhstan (44°35′ N; 76°66′ E). The *P. caeruleum* plants of accession K 3345-02 grew in a natural habit in the Moscow region, Russia (55°72′ N; 37°23′ E).

### 4.2. Sequence Analysis and Identification of DNA Repeats

The genomic DNA of *P. caeruleum* cv. ‘Lazur’ was isolated from young leaves using the CTAB method with minor modifications [63]. Genome DNA low-coverage sequencing was carried out with the use of the SURFseq 5000 sequencer (GeneMind, Shenzhen, China) according to the NGS protocol for generating 25.9 million of paired-end reads of 150 bp in length, which was 0.66× of the coverage of the *Polemonium* genome (1C = 5916.9 Mbp) [64]. Due to the limited information on the genome sizes of the *Polemonium* species within the Plant DNA C-values Database [65], we used the genome size data of the only species available in the databases, *P. reptans* L. (6.05 pg), which contained the same chromosome number (2*n* = 18) and a similar chromosome morphology [64,66].

The raw data were uploaded to the NCBI database (https://www.ncbi.nlm.nih.gov/sra/PRJNA1277527 (accessed on 18 September 2025)).

In addition, for genome-wide comparative analyses, the publicly available *P. caeruleum* sequencing data (ERR5555406 and ERR5555143 samples, project PRJEB43865, https://www.ebi.ac.uk/ena/browser/view/PRJEB43865?show=reads, accessed on 9 April 2025) were used. The collection site of sample ERR5555406/SAMEA8202199 is located in northern Norway, west of Sifjord (latitude: 69.2842; longitude: 17.1232). The collection site of sample ERR5555143/SAMEA8202365 is located in northwestern Norway, near Bostranda beach on the west coast of the island of Senja (latitude: 69.4704, longitude: 17.2323).

The bioinformatic analysis of the *P. caeruleum* repeatome was performed using RepeatExplorer2/TAREAN/DANTE_LTR pipelines based on the Galaxy platform (https://repeatexplorer-elixir.cerit-sc.cz/galaxy/, accessed on 26 April 2025) [67,68,69].

For each studied sample, the genomic reads were filtered by quality. Then, 1,500,000 high-quality reads were randomly selected for further analyses, which corresponded to 0.04× coverage of the *P. caeruleum* genome (1C = 5916.9 Mbp) and is within the limits recommended by the developers of these programs (genome coverage of 0.01–0.50× was recommended) [67]. RepeatExplorer/TAREAN was launched with the preset settings based on the Galaxy platform 2. The sequence homology of the identified tandem DNA repeats was estimated using the Basic Local Alignment Search Tool (BLAST) (NCBI, MD, USA). Seven identified abundant tandem DNA repeats of *P. caeruleum* were used for generating oligonucleotide FISH probes (Supplementary Table S2) using Primer3-Plus software (version 4.1.0) [70].

### 4.3. Chromosome Spread Preparation

The chromosome spread preparations were made according to a previously described technique with minor modifications [27].

The seeds of the *P. caeruleum* were germinated in Petri dishes for 3–5 days at room temperature (RT). Root tips (5–10 mm long) were cut off and incubated in ice water for 24 h for the accumulation of mitotic cells. After that, the root tips were fixed in ethanol/acetic acid fixative (3:1) for 48 h (RT). The roots were transferred into 1% acetocarmine solution in 45% acetic acid for 20 min. Each root tip was placed on a glass slide; the meristem was cut off, macerated with a dissecting needle in a drop of 45% acetic acid, covered with a cover slip, and squashed. Then the slides were frozen in liquid nitrogen, dehydrated in 96% ethanol, and air dried.

### 4.4. FISH Procedure

In FISH assays, two wheat DNA probes, pTa71 containing 18S-5.8S-26S (45S rDNA) and pTa794 containing 5S rDNA, were used [71,72]. These DNA probes were labeled directly with fluorochromes Aqua 431 dUTP and Red 580 dUTP (ENZO Life Sciences, Farmingdale, NY, USA) by nick translation according to the manufacturer’s protocols. Moreover, we used oligonucleotide probes Pol_C 1, Pol_C 33, Pol_C 46, Pol_C 67, Pol_C 70, Pol_C 125, and Pol_C 134, which were synthesized and labeled with Cy3-dUTP and/or 6-FAM-dUTP in *Syntol* (Moscow, Russia) (Table S2).

The FISH procedure was carried out following a previously established protocol [73]. Briefly, before the FISH procedure, chromosome slides were pretreated with 1 mg/mL of RNase (Roche Diagnostics, Mannheim, Germany) in 2 × SSC at 37 °C for 1 h. After three washings in 2 × SSC for 10 min each, the slides were dehydrated in the graded ethanol series and air dried. Next, 40 ng of each labeled probe was dissolved in the hybridization mixture (contained 50% formamide, a total volume 15 μL) and dropped onto the slide. Then the slides were sealed with rubber cement under coverslips, co-denatured at 74 °C for 4 min, and hybridized overnight at 37 °C in a moisture chamber. After the hybridization, the slides were washed in 0.1 × SSC and then in 2 × SSC (for 5 min at 42 °C each) followed by a 5 min wash in PBS at RT, dehydration in the graded ethanol series, and air drying. Finally, the slides were stained with 0.1 μg/mL DAPI (4′,6-diamidino-2-phenylindole) (Serva, Heidelberg, Germany) in Vectashield mounting medium (Vector laboratories, Peterborough, UK).

### 4.5. Analysis of Chromosome Preparations

At least five plants of each accession and fifteen metaphase plates from each specimen were examined. The chromosome slides were analyzed using the Olympus BX 61 epifluorescence microscope equipped with a standard narrow-band pass filter set (Olympus, Tokyo, Japan). Images were acquired with a monochrome charge-coupled camera (Cool Snap, Roper Scientific, Inc., Sarasota, FL, USA) and processed using Adobe Photoshop 10.0 software (Adobe, Birmingham, AL, USA).

Chromosome pairs in karyotypes were identified according to chromosome size and morphology, as well as the localization of the chromosome markers. In the karyograms, chromosome pairs were set in decreasing order of size.

## Figures and Tables

**Table 1:** Proportions of the major DNA repeats identified in the genome of *Polemonium caeruleum* using RepeatExplorer2 pipelines.

Repeat Name	Genome Proportion (%)
Retrotransposons (Class I)	66.08
Ty1 Copia	19.25
Ale	0.24
Ikeros	0.06
Angela	7.66
SIRE	10.70
TAR	0.31
Tork	0.28
Ty3-Gypsy	44.46
non-chromovirus Athila	10.90
non-chromovirus Tat- Ogre	0.86
chromovirus CRM	0.40
chromovirus Galadriel	0.06
chromovirus Tekay	32.19
chromovirus Reina	0.05
LINE	0.08
Unclassified LTR elements	2.29
Transposons (Class II)	0.57
Cacta	0.17
hAT	0.04
MuDR_Mutator	0.36
rDNA	0.42
Unclassified repeat	3.31
DNA satellite	0.87
Repetitive DNA	71.25
Putative satellites	6 high confident 3 low confident

**Table 2:** Homology of the tandem repeats identified in the genome of *Polemonium caeruleum*.

SatDNA/Genome Proportion, %/Repeat Length, bp			Blast Homology
‘Lazur’	ERR5555406	ERR5555143	
Pol_C 33/0.44/508	CL67/0.42/509	CL59/0.54/411	93%/92% of coverage/identity with CL67 in ERR5555406 sample 99%/92% of coverage/identity with CL59 in ERR5555143 sample
Pol_C 46/0.24/191	CL90/0.18/192	CL86/0.22/191	99%/100% of coverage/identity with CL90 in ERR5555406 sample 95%/100% of coverage/identity with CL86 in ERR5555143 sample
Pol_C 67/0.12/89	CL84/0.22/89	CL89/0.2/90	100%/100% of coverage/identity with CL84 in ERR5555406 sample 79%/100% of coverage/identity with CL89 in ERR5555143 sample
Pol_C 70/0.092/393	CL124/0.05/393	CL117/0.062/393	100%/98% of coverage/identity with CL124 in ERR5555406 sample 100%/98% of coverage/identity with CL117 in ERR5555143 sample
Pol_C 125/0.016/83	no	no	no
Pol_C 134/0.014/364	CL159/0.022/364	CL152/0.021/364	100%/100% of coverage/identity with CL159 in ERR5555406 sample 100%/99% of coverage/identity with CL152 in ERR5555143 sample

## Data Availability

All data generated or analyzed during this study are contained within the article and Supplementary Materials.

## Supplementary Materials

The following supporting information can be downloaded at: https://www.mdpi.com/article/10.3390/ijms26189240/s1.
