# Supplementary material for: Integration of Repeatome and Cytogenetic Data on Tandem DNAs in a Medicinal Plant Polemonium caeruleum L
Source: Int J Mol Sci. 2025 Sep 22;26(18):9240. doi: 10.3390/ijms26189240 (PMC12470644; doi:10.3390/ijms26189240)
Supplement: Supplementary file 1 [file ijms-26-09240-s001.zip › ijms-3824277-supplementary.pdf]

*Supplementary Material*

**Table S1.** Characterization of the tandem DNAs revealed in repeatome of *P. caeruleum.*

***P. caeruleum* putative satellites (high confidence)**

| Tandem Repeat/ Genome proportion [%] | Repeat length, bp | Sequence |
|---|---|---|
| Pol_C 33/0.44 | 508 | ATTAAAGTAACTATTTTCATCCAAAATGTTATAGTTTACAGCCATAATGTTTACA CTTACCTCCACAACTTCACATAGAAATTGAAGCATGATGTCATTGTGAATTTAA TTCATTGCAACATGACATAAGTTGTTTTGCAACATACCTTGTGACATTTACTTGT ACAATAGTGAACATTTAATTCAAGGGGTGTTCACCACCACCACCTATCGATGTA TCTCATTCACCAGTATGATACACTAAACCTCAAGCATCAACAACATGATGTCTT TGGCCTAAGACATGGACTCTCATTTGCATTCCATAGGCATTGTATGATCTAAGG GATTCACTCCTATGTTTACATGTGCAAGGGATTCTCTTATGCCAACACTCTAGAT GAATGTTATGGTTTTCATCCACTTAGGGTAAATGTGTTTCCATCTAATTTGGTGG CCAACATGTTGGTGATTTTTACTCCCTTACGTTAGGAACAACATCAAGGCATTC AATTAAACAAAAAAAAAC |
| Pol_C 46/0.24 | 191 | ACTTCTAATAATCCATATTGGTCCCACTTCTATTCAAAATTCAAACTATCTATAT AACAATCTACTATGGGGATTAGTGTATCCCCAAAATATGACAAGTGGAATAGT TTATCCCGCCTCTTTAATTTACAGTGTAAACCCTTGAATGTGTGGATGAGAAATT TAGAAATCCAAACAATACTCTTTTTTT |
| Pol_C 67/0.12 | 89 | CTTTTGAGCCCAAACAAAAAATAAACATGTATTTTTGAAAATAAAAATGATAA ATATCTTACTAATGGGTCATTAATTTCAATGGGCCT |
| Pol_C 70/0.092 | 393 | ACCGGGATGGTCAAATGAACATCACAAATATACCTACCTTCGCATATTGTTAGG CTAGGAATCACCTAATACATGTTTTAATATCCGTTACTTAGTACGTACAATGTG GGAACATAAAAGTCCATAGTGAGGTATGAAATAAATGAACATAAATATAAGC CATTCGTGCGTTTCGTAGTCCCACACTACCCTTTCTTATGTTTCTATAACCATTAC GTAGAATGTCCATGTGTATAAAACTAGAATCTATCTCACAAGAAGGACTATAA TGAAAGTTATGTGACAACACTAAACTAAATTAGTAATAATTACAAGTCACATA TTTGGTGGTGTTAGGATCTATCGATTCTTAACCCCACATGCATGAAGTTGATAA CTTGTACGACGATATGT |
| Pol _C 125/0.016 | 83 | GTAAGTTCACATATCTCACTACAAGCTAAGAGAGAACTCCATGACTATTAACGT CCAAATACAACTACCACTACTACATTTCA |
| Pol _C 134/0.014 | 364 | CCGAGACTTCCATGTGCAAGTTGACACGGGGAGTGATCTACTCTGGGTTAATAG TGTTGCATGCACTGGCTGCCCTCACGTGTCAGCTCTCGATGTGAGTACACTATTT TCTAGTCCGTTGTTAGGTTTTTAATCAGTTGTATTTCTTTGCTTATCATTATTAAA GCTATTTAAATTATACAATTAATGATAAGTGGCTTTGAAATTTCAGATTGGACTC AATTTTTTCAATACCTCCAGTTCATCGACGGCCTCATCAGTGTTCGATAAATGA AGATGTTGATTTAATTAATTATTTTTTGCGATATTTGGAATGGTTTTGCTGTAGA ATTTACTACACAAAGATTGCACTGGGCTCGCCACC |

***P. caeruleum* putative satellites (low confidence)**

| | | |
|---|---|---|
| Pol_C 140/0.012 | 187 | TCTACTACTAAAAAGAATGAAGAGTTCCTAATATTTTCTGTAAGGACAAAAAT AGCCCTGAACAATCAACATATATTTTTAATGAATGAATGATGGTGTTTTTAATCT CTGCTCTTTAAATGAATGAAGGTGTTTCTAATGGTTCCTAATATTTTTAAGACAA AAATACCCTTAAACAATCTCTATC |

| | | |
|---|---|---|
| Pol _C 142/0.012 | 168 | GTGGTACTAATTAATCATAGCTAATATACATATGTACCAATTTCAAGTAACCGG<br>TGTGACCTCTCCGGTCAATTGGTGATCGACTGAGACTATCGGTTCAATTTGACT<br>AGTAACCCTATTTTGGGGACAACGACCGAGATAGACCAATTAAGTCAATTGGA<br>GTTGTAA |
| Pol _C 158/0.01 | 523 | AATTTCAATCTAAACCACCTTGTAAGAACCCTCGGATCAATAGGAGTTCGACTG<br>TCACACTCGGTCCAATTTAACTTTTAACCTCGATCTGGGGTTAACGACCAAGAG<br>ACACTAATTTCAACTATTAAATTTTATTATGTTATTTATTACGCGTTGAGTTATGA<br>CATATATTCTAATTTTAACAACATTGACATACTGCGTAACGGACATTGAAACAT<br>ATAAAAGGGGTATGTCAGACTACGAAAAGGCAATAGTTAGACTACGAAAAGG<br>CAACAATTGGACCTAGCGGTCTTATAACTATAATTAGACATGTAATGGCCACTA<br>AATGAGTTTAATAGTGTTACATATCCTCCAATTTAAAACATCTTGTGAGACCGC<br>TAGGTCCAATTGATGTTCGGTTGACAATCCCGGTATATTTTGACTATTAATGTTT<br>TAAACGTAATATGGGTTTAACAACCGATAGAGACTAACAGTACCAATTTGACT<br>TGTAACTATTACTAATTTAGTGTAGTGTTATTACATA |
| | | ***P. caeruleum* putative LTR** |
| Pol _C 1/10.0 | 9509 | GATGAGTCTTTTCTCTTCTGACTCATACTACCTGTCTTTCCACCAACCTGAATTCT<br>TTGTGATTTTCCTGATGTCATCTGGTTAGGGTTTAGGGATGAGAGGTTTGAGAA<br>AGAGAGAGATAAAGATTTTCGTAGAAGAGAGGGAGTAGAATGATTTGAACTTC<br>AGAGAGTATGGAAGAATAGTTTAACTCAAACTCTTAAAATACTGATAGAGTTTT<br>GAATAGTCAAGATAATTTCTGAGATTTAAAATTTAAAACTTAAGAGTTGACAAC<br>AAATTTGGATAGATAGTATATCTTCAGAATGAGATAATAACTGAAAAAGATCC<br>GAAATATTAGAGATATGGAGAGATTTTGGCAAAAATAGATCTTTACAAGATTTT<br>CGGAGATTTTAAATATTTAAAAATTCAAATCTTTAAATTTTCGACAGTTGAGAG<br>ATAGAGATCACATCATTACCTAAAGAGGTAACACGCAACCTACAAAATTGATC<br>CATAAGTTTACTGAATTTGGCATGCAGATTTATTCCCAAGGCCTTGAGAACTGG<br>TCTCAAGGTCTTGAAAGACTGTTATGCACAAAAACACATAAGTCAAGGTCTTG<br>AGCTGAATTGCCAAGGCCTTGACAATACCTGATTTTTTCTGATGCTCCCCCTAA<br>ATCACAGCATCTTTGTTAATCATTCCAAGTTCCAATCTCAAGCATGTAAATCTTT<br>CATCTGGGAGAGGCTTTGTGAAGATATCTGCAATCTGAAAGTTAGTAGACACA<br>AATTCTAAACAGATATCACCTTTTTCTACATGATCCCTGATGAAATGATGTCTA<br>ACATCAATGTGCTTTGTCCTTGAGTGTTGGACTGGATTCTTTGAGATTGATATTG<br>CACTTGTATTATCACATTTGATTGGAATGTCTTTGTAATCAAATCCCAAGTCTCT<br>CATTTGTTGTGCAATCCATAGAACTTGTGAGCAACAAGCTCCTGCTGCTATGTA<br>TTCAGCTTCAGCAGTTGATAGTGCCACTGAGTTTTGCTTCTTAGAATGCCAACTG<br>ATTAGTGATGATCCAAGATATTGGCAGCATCCTGAAGTACTCTTTCTATCAACTT<br>TATATCCTGCAAAGTCAGCATCTGAATATCCACATAGAGTTAGTGCAGTTTTCTT<br>TGGATACCATAATCCTAAGTCCTTTGTTCCATTTAGATATCTCAGTATTCTCTTC<br>ACAGCTATTAGATGAGATTCCTTAGGGTCAGCCTGAAATCTTGCACACATGCAT<br>ACACTAAACATGATGTCTGGCCTGCTTGCAGTTAAATATAGCAATGATCCAATC<br>ATACCTCTATACTCCTTCTGATTAACACTCTTACCAGATTGATCCTTGTCAAGAG<br>ATAGTGTTGAACTCATAGGGGTTTTTGCTATTTTGCATTGAGACATGTTATACTT<br>GTTCAATAAATCTCTTGTGTATTTGCTCTGGTGGAAGTAGATTCCATTCTTCAAT<br>TGGTGCACATGTAATCCAAGAAAGAAATTCAGCTCTCCCATCAGGCTCATCTCA<br>AATTCCCTGCTCATAAGACTTGAAAATTCCTTGGCCAACAGATCATTAGTAGCA<br>CCAAACAATATATCATCAACATAAATTTGTACTACTAATATGTCTTTTTCATGAG<br>TCTTAACAAACAGTGTTTTATCAACAAGTCCTCTTTTAAAACCATTTTCTAAAAG<br>ATGTTTTGATAATCTTTCATACCAAGCCCTTGGTGCTTGTTTCAAACCATATAAA<br>GCTTTATCCAATTTATAAACATGGTTTGGAAACTCATCATTTTCAAATCCTGGAG<br>GTTGAGCAACATATACTTCTTCTTGTAGATATCCATTTAAGAAAGCACATTTGA<br>CATCCATTTGATATAATTTCATTTTCTTATGAGATGCAAATGCAAGAAGTATTCT<br>AATTGCTTCTAACCTGGCAACTGGAGCAAATGTTTCCTCATAATCAATTCCTTCT<br>TCTTGGTTGTAGCCTTGAGCAACAAGCCTTGCTTTGTTTCTCACTACCTTACCATT<br>TTCATCACATTTGTTCCTGAACACCCATCTTGTACCAATTACTGACTGATGCTTG<br>GGAGCTGGAACCAGGTTCCAAACCTTGTTCCTTTCAAACTCATTGAGTTCACAT<br>TGCATTGCAATAATCCAGTCAGCTTCCTGTAGTGCTTCTTTGATGTTCTTTGGCTC |

| | |
|---|---|
| | AATCAATGATACAAAAGCATTGAAAGCACACAGGTCTTTCAGTTGTCCTCTGGT<br>TTTGATTCCTGTCTTTGGATCAGAGATGATGTTCTCCACTGGATGTGGTGCTATG<br>AGTCTTGGCCTTTTCCTTGGCATTCAAGGTTCTGGTCCCATAAGAGATTCTTCTG<br>ACCTTTGAGAGGTGGAAGTAGTGGGTTGACCACTTTCTGATCTTTGAGGTTCAC<br>CACCAAGGCCTTGAGAATTGCTGTCAAGGTCTTGAGAGTGCTCTCCAAGGCCTT<br>GAGCTTCCTTGTCACCATCTTCTTTTTCATCATCAGACTCATGCCTGTTTAAATCA<br>AGCTCTGCAAGACTTTCACTCAGAGAATCATTAGTACTTTCATCAAATACAACA<br>TGGATGCTTTCTTCTACTACTTTGGTTCTTTTGTTCAATACTCTATATGCTTTGCTA<br>TTTTGAGAATAACCTAGAAATATTCCTTCATCACTTCTAGCATCAAATTTTCCTA<br>GGTTATCCTTTCCATTATTATGAACAAAGCATGTACATCCAAAGGGTTTGAAAT<br>ATGAGATATTTGGTTTTCTGCCTTTGAACAATTCATATGGGGTTTTCTTCAAAAT<br>AGGTCTAATAAGGCATCTATTCAAAATATAGTTGGCAGTGTTAACAGCTTCAGC<br>CCAAAAGTATTTTGGTAAGTTATTTTCCAAAAGCATTGTTCTAGACATGTCTTCT<br>AGGGTTCTGTTTTTCCTTTCTACAACTCCATTCTGTTGGGGGGTTCTAGGTGCAG<br>AGAAATTATGTGAAATTCCATTTTTCTCACAAAACAAATCAAATTCAAATTGAT<br>CAAACTCTCTGCCATGATCACTCCTGATGGAAGCAACAGGCAGGTTTTTAGCAT<br>TTTGTAATTCTTTGCACAGTTTTGAAAATTCACCTAAGGCCTCAGATTTATCCTTT<br>ATAAACATGACCCAGGTGTACCTAGAATAATCATCTACAATTACTAGCATGTAT<br>TGTTTACCTTTAGGAGTTTTAACCCTCATAGGTCCACACAAGTCCATATGCAAC<br>AATTCAATGGGTCTAGAAGTACTTACAAACTGTTTTGGCTTGAAAGAACTTCTT<br>GTCTGTTTACCTTGTGTGCATGCCTTGCAAACCTGATCATCTTTGAATTTTATAG<br>AGGGTAATCCTCTTACCAAGTCTTTGGACACAAGCTTGTTGAGAGTGTGTAAAC<br>TGATATGGCCAAGTTTCCTGTGCCAAAGCCTTGAGTCATCCTCCAAGGCCTTGA<br>GGCACTTCAGCTTCTCACCTGCAATCTTGTTAGTATACAACTCATATACATTATT<br>TGACCTTTGTGCTGTGAGAATGCATTTTCCTGATTTTTCATCTTGTACCACACACT<br>TCTCTTTGTCAAATATTACCTTGTTGCCTTTGTCACACAACTGTGAAACACTTAA<br>CAAATTGAACTTTAGACCATCAACAAGATACACATCAGATATAGCATTTGCAG<br>TTTCATTTCCAACTTTACCAATACCTACTATGTTGCACTTTCCTTTGTCACCAAGT<br>GTGATTGTTCCTCCAGTCTGTTCCTTAAGTTCAGAGAACAAAGAAATCTTGCCA<br>CTCATATGCCTGGAACAACCACTATCCATTATCCACTTTTGTTCTTTATTCTTGGT<br>GACAGCCTGCAAGAGATGATCAATGCCTTACCCAGCTTTTAGACCAGGTGTTA<br>AACTTCACATCATATTCATCATCTAACATATTAAGAGGTCTAGTTCCTTTCTTAA<br>CCCATATTTGCTTAATTTCTCCTTTAAGTCTAACTGGAAAGTTGTTCCTCATAAT<br>ATGTGGTTCTGTTCTTCTAAAAGGACAAGTGAATTTGACATGACCAACCTTACA<br>GCAGTAAAAACATGTAGGAGTGTTTTTATTCTTAGGCTTCACAAAAACAGTGTT<br>GGATTTCCTTGGAAAATTAGGGTTGTAGCCAATGCCATGCTTATGAATAGATTG<br>GGTGCTTTTGCTTAACATCAAATCTAACTTCTTTTCTGAGCAGTTAAACTTAGCA<br>ACCAATTCCTTAGATTCTTCAAGCTGTTCAGTCAATGCAACATTATGTTCTTCTA<br>TTTCCTCATGCTGAATCTTTAAGGCAGTAAAGTTATCACTAAGCAAAGAGTATT<br>CATCAATTACTTCTTTGACCTGAGTCTTCAAGGTCTTGAAATTGTTTTCCAAGGC<br>CTTGAGATCTCTGTCAGACTTAGCTTTAGATCCTTCAAGAACTTTGTTTTGATAA<br>ACTAAATCTAGATTTTCCTTGGTTTGCTCATGTAGTTTAACTTTCAAAGTTTTGAT<br>TTCATTCAGATAACTTTCCTCATTCTTAAACATATTTTTCAGCATTTCAGATAGA<br>ACATCTTTAGGACTGGAAATGAGGTTGTTTACCATTACCTCAATGTGTTTGTCTG<br>AACTTTCTTGTTCAGCCATCAAACACAGGTTGGCTTGTTCTTCTTCAGATTCTGA<br>TTCATCTGAGCTGTCTGAATCACTCCAGGAAGCCACCATTGCTTTCCTTTTGTCC<br>TTATCTTTCTTATAATGTTGCTTTTTCTTCTGTGGACAGTCTTTCACCAGATGATC<br>TGTTTCACCACATTCAAAGCATTGCATTAGTTTAGATTTATTGAAATTGGAGTTA<br>GGCTTACCTCTGTGATCCTTGCTTCCTGAGGATCTTGGTCTGTCTCTGTTGTATTC<br>CCTGTTCCTGAAGTTCTTCTTCATCTTTAGAATTCTGGTCAAGGCCTTGGTCATG<br>AGTGCAATTGGATCTTCATCATTTTCTTCATCTTCCTCAAGACCTTGGCTTTGATC<br>AGTGTCTTTAGCTTTGAGTGCTAAGTTCTTCAGGTTGTCTGAGAGGTCTCCATCT<br>CCTTCATTCATTGACATCTCATGTGTCAATAGCTTCCCTTGTAGATCATCTAACT<br>GGAGTATGGATAGATCTTGAGCTTCCTCAATTGCTGTGACCTTAGGATCCCATTT<br>AGCTTTAGGAAGACTCCTCAATATCTTTCTGACTTTCTCTTCATCTGAAAAGGGT<br>TTTCCAAGAGACTCCAGATTGTTTACAATGTCAGTAAACCTCTTGAACATGTCTT<br>TGATGGATTCTTCTGCTGTCATCTTGAAGAGTTCATACTGTCTCAGATAGATATT<br>GATCTTTGTCTGCCTCACTTGACTTGTGCCTTCAAATGTAACAACCAATTTGTCC<br>CAAATTTCTTTGGCACTTTTGCAGGTTGATATCCTGTTGAATTCATCAGTACTAA |

| | |
|---|---|
| | GAGCACAATATAAGTGGTTCATAGCCTTGTAATTCTTGGCTATCTTCTCAAGCTC |
| | TGTGGATGTGAGATCTGCCTCAGTCTTTACTGTCCTTACTCCATCTTCTGATACA |
| | GTGTGATAGACATGAGGTCCATTTTGGATGATAGACCATAGCTCTATATCTTGA |
| | TCAATGATGAACATCTTCATCAGGGACTTCCAATGCATGAAATTGTCTCCACAG |
| | AATAGTGGAGGCCTGTTTGAAGCTCTGCCCTGTGAAAATCCTCCATCACCTACA |
| | AAACTCATGATCACCAGAATGTTAGTTCTCTACAGTGATACAAGCTCTGATACC |
| | AATTGAAAGTTGAGTAGTGTAGGTGTGTATAAGTGTAACTATACTCTAGAAGGG |
| | GGGGTTGAATAGAGTATGCCCAAGTATGCCCAATTTTTGTTTGTTTCAAACTTGC |
| | GGAAAGATTTGTAAATGCTCAAGGCCTTGGGAAATCAGTTTGTCAAGGCCTTGA |
| | CAAAATCAGTTTGAAAGCAATGAACAAAACAGAAAGTAAATGAGACAAGACA |
| | CAGTATTTGTTTCGAAGTTCAGTCCCAATGGACCTACTCTCCGCCTGAGGGATC |
| | CAATCCCTCTAGGATTTCAAAACCTTTCTTAAATATCACCAGCCAAACCCCTAG |
| | ACTTACAGACCAAGCAGAGTTCCCTTACTACTTGTCTGTGGATAATCCTATCCCT |
| | CTAGTTACAGTACTGACTTTGATAAACTAAGGACTCCTAATTACTCCAATGAGT |
| | AATCCCAGCTGTTCCAATGAACAACCTTACAACCACTGAATACAGATAAATGA |
| | ATACAGTGATAGAATAGAAATGAAGAACACAGAGAATGGATAAGCCAATGAT |
| | AGCTTGGATGTGTTCTCCTTGATGTTGATTGAGATGTCTGGATTTCCTTCAATGT |
| | AGATCACTCAATCTGACTGAGAATGGATTTGCTCCTCTGTTAGCTCACTCAGTCT |
| | TGATGATGAATAGCTCTTGGATTTTCGTGTGTAAGCTCTCTGTTGTGTAATGTCT |
| | CTGTGTCTGTAATTGTTGTCTTCAGCCTTGGTGAAGATCCTCTTTTATAGCTTCTT |
| | CAAACTAGCCAGTAGATAGCCAGTAGACAGTTGCAACAACCTTCCTAGAAATC |
| | CTCCAGCATCTTCATCCTCTCAACAGCTTGATCTCCAACGGATCTGTGCTGTTGT |
| | AGACTTCTAGAATCTGATCCTTGGCTTGATTTTATCCTCTGTGAATATCTTGGAC |
| | CAAATCCCTTGAATCTAGGTCTGTTTGAATTCAATATATTGCACCTGCAATTTCA |
| | AACAGCACAGTCAGTGAACCTCAAGGCCTTGAGACTAGCCTTCAAGGTCTTGA |
| | CAGTTCTGAACCAAAGTTCACTTCCTGTCAAGGCCTTGAACTTCAAGTTCAAGT |
| | TCTTGATCCTTAGCACTGTATTAGTATAATCATGATCTCTTAGACTTGTATTCCAT |
| | CCTGTATACACCATATGTTAATAGCACAATATAAGCATAGTATAACAGGTTATC |
| | TAGATCATTTAGGCTTATTATCTTTCATTATCCTGCATATACATTCTGTAGTTAGC |
| | ATAGCATGAGCACAGTATAGCAGATAATGTATTTCATTCATGCCTTTTATATTTC |
| | ATCCTTCACACAGTATGATATACTCTCTTTTATAACTCATATATCCTGTATCATCT |
| | ATATATTCTATATAATGTAAGTCCATGATGCACAGGTATAAATGTATCATACTT |
| | AATCATTTGTCTTTCATATTTATCTTTCATCACCATAAACTTAATCATTTGTCTTT |
| | CATCATCCATCACATAATTCATCACATAAGCACAATATGATCAATACCAAATA |
| | GATGAATCATGTTAACAGTTATAGTCAATATCAAATAGACAAATCATATAAGC |
| | AGATATAACAGATATAACATATTAAGCACATAAGCAGATATAGCTTTAGGCAT |
| | TGTCAAGGCCTTGGCCTTGCTCCCCCTAAGCTTATGCCTTGTCTTATTTCATCAA |
| | GGCCTTGGGATTGACTTAACAATCTCCCCCTTGATGAAAGACAAAACCAAGTT |
| | ATGTTTATGCTTCTCTGTATCAACCTGTGCTGCACTTAAGATCAACCAAATGTTA |
| | GTACAGTTTATGATATGGAAAGATAATGTAATAACAGATATTGAAACCAAAAT |
| | CCAAATCACATAAACTGGTCTATGTGAGCACCAGATCAAAAGAATAATTATCC |
| | CCTTACACTGGCCTAACTAAGCACCAGTACACCAACTAAGCACATAAACTTCT |
| | CCCCCTTTTTGACTTTCATCAAAAAAGTGAGAGAAAATGTTGGATCAGAAAATT |
| | TATTACAGAAAGATTGTTTTCAAAATGGACTGAAAAATTTAAAACAGAAAGAG |
| | ATTTAGAAGAAGTGGAGAAGATGGAGAAGAGAGGGTAGGGTCAAGGTCTTGA |
| | GGGTTAGTCTTCATCATCCTCAGCCTCATCAGATTCCTTATCAGCTGTTGTCTCTT |
| | CTTCTTCCTCTGTTTCTTCTTCATTCTTTTCCTTTTCCTCCTTTTCCTCTTCATCCCC |
| | TGAGTCCTCAGCATCACTAGCATCCTTTTCCTCTTCCTCAGCATCAACTGAACCT |
| | TGAGCAGTTTCACCACCAGCATCCTCAAACAGGTCTTCTGAAGTTACAGGTGCA |
| | GGAGCTGTCTTCCTTATCCTGATCAACCTCTCTGCCAGAGGAATGTCTGACTCTA |
| | TGCTATCATCATCTTTTTCCTTATGCTCAGAATCCTTACTTGGATCAGCATTGAG |
| | ATTCTCTCCAAGTGATACTATAGGAGAGCCTGAAGGTTTTGCCACATTCTCCTC |
| | AGAACTGGTTTCTTCCACCTTCAAGGCCTTGGAGATTTTGTTCACTTGGCTATTC |
| | ATAGCATTCAGTAAGAACTTCATCTTTCTGAACTCAGTACATAGCTCAAGCTGC |
| | CCTTGCTTAAGCTCATCAATACCAAACTCAATTGATTCCAATCTGTCTAGAGCA |
| | AAAGATGAGTCTATAGGAATAGATGGTGAGGGTTTGGGTTCTGTCAAGGTCTTG |
| | AGTCTGGCAAATTTCTTAATTTTGATTTCCTCAACAGGTACACTCTTCTGAAATT |
| | TCCACTCATCCTTCACAGTTCTTACTATTCCCAACCTCTGCAAGGTAATCTCATT |
| | GATTAATCCTATTCCAAAACTTATCTTCTCTTCCTTTTTAACAGAAATCCCAAAA |

| | | |
|---|---|---|
| | | TGTTTGAAGAACAAGGGTAGAAGATTGGCATAAGGTAGTATGTGGTTTCTCTGG<br>TGGGTTGAAGAGTAGAGCATGTGTTCCATTACAAGCTCTGGCAAGTCTATCTCA<br>TACCCCTGCATTATCAAATGCATCACATATAACACCTTGTCAGATAGCAGTTCC<br>CTTGAGTTTTCTTTTGGAATCACACATCTCATCAGAATGGTAAACAGTATCTTAG<br>CTTCTACAGTGAAGACTGTGTGGGTTAACTGTTTGTTGCTAGATGTGGAGGTGT<br>GGTTTGGTAGGAAAAACTCAGATTTTGCTAACCTAATCCCTTCCTTCATCTCAGG<br>GTGATCATCTACCTTATAGCTTTCAGGACAGCCCAAGACCTTGGCAAATACAGA<br>ATGGTCTAGGATTATGTCCTTCCCTGCTACCCTGGAGGAGATAACCAACCTGTC<br>TCCTTCCACTACTTGAAGATTGAGGTAAAATAACCTAGTTAACCTAGGATATCC<br>AATAACACCAGGGTTGATTACCTTCCATATTGAACTCTTCTTCATCTTTTTCATG<br>ACTTCAAGATACATATCCTCAATCAAGTTAGGACAAATCATGCTACAGAATGA<br>TATAGGTTGTAAAGCATAACCATCTAACCACCTTCCTCTAGCAGTATGATTAAT<br>AAACCATTTAGCAGGTGCAGAGGAATTCTTACCAATTTTCTCCTTTGGTACCTTC<br>TCAGTTGTTTCTGTTTTGCCCTTATCTGATTCCCTGAGAGTCTTAACCTCCTTGAT<br>CTCCTTCTTTTTCTTGTCCTCTATCTTGGAACTGCCTATTCTCTCCTCATTGTCCTG<br>CTTCGATTTCTTTGGATGAGAGTTTATAGTTTCAGTTTGCAGGTTCACATTGTCTG<br>TGATGATCTTCTCTACT |
| Pol _C 2/7.5 | 5579 | GCCCGGAGTCCTAAAAGGAAGAGCAAAGTGTTTGCTAATCCTAAAGAGCAAA<br>ACGTTTGCGCACCCTAAAGGGAAGAGCAAAAGTGTTTGCAAAACCCAAGGGT<br>AATGCCTTGAAGCCTATTGGTGGGGTTGCTATGGGCAAGTGTCACTATGGTAGT<br>GGACCAGGCCACTGGAAGAGAAACTGCCCAAAATATCTGGAAGACAAGAGGC<br>TCGGAATAGTGATTCCACCACCTTCTAGTATCTTTGTTATAGATGTCAACTTGTC<br>TATTTCTTCATCATGGGTATTGGATACCGGATGTGGATCTCACATTTGTCGAAAT<br>GTGCATGATCTGAAAAAGATATAGACGATTGGCTAGGCGAGAGATTGACCTAC<br>ACGTTGGAAATGAAGCAAAGTGTTGCTACATTGGCCGTAGGAACTTACGAGTT<br>ATCTCTGCCTTCTGGACTTATCTTAGAATTAGACAATTCCAATAATGTACCTGCT<br>ATGAGTAGGAACATTATTTCTATTTCTTGTTTGGACAAGAAAGGATTTTCTTTCA<br>TAATAAAGGACAAGTGTTGCTCCATTTATTTTGATAGTTTGCATTATTGTAATGC<br>GCTTTTGTCTAATGGATTATATATCCTTGATCTAGATATTCCAGTCTATAACATA<br>AATACTAAGAGGATTAAATCTAACGATTTAAATCCCACGTACCTTGGCATTATC<br>GTTTGAGCCATATAAACAAGAAATGCATTTCTAGACTCCATAAGGATGGACTTC<br>TAGACTCATTTGATTATGAATCATATGAGACATGCGAGGCTTGTTTATTAGGTA<br>AGATGACTAAGTCCCCATTCACTAAGAGTAGTGAAAGGGCGAGTGAATAATTG<br>GCACTCATACATACTGATGTGTGTGGACCAATGACTACTCATGCCAGAGGTGGT<br>TATCCATACTTCATTACATTTACTGATGACTTCAGTAGATATGGATTTGTGTATC<br>TTATGAAGAACAAATCTGAATCCTTTGAAAAGTTCAAAGAATTCAAGAATGAA<br>GTGGAGAACCAACTTGGGAAAAGTATTAAGGCTCTACGATCGGATCGTGGAGG<br>AGAATACTTGAGCCAAGAGTTTGACGATCATCTTAGAGAGTGTGGGATCTTGTC<br>CCAACTCACTCCACCTGGAACGCCACAATGGAATGGCGTGTCTGAGAGGAGGA<br>ATCGAACCCTGTTGGACATGGTTCGATCTATGATGAGTCAAACAGATCTTCCGA<br>TATCCTTTTGGGGACATGCTCTATTGACAGCTATCTTTACCCTTAACCGTGTTCC<br>ATCCAAATCAGTCATGAAGACACCATATGAGATATGGAGTGGGAGGCGTCCCA<br>ACCTGTCGTTCATGAAGATTTGGGGTTGCGGAGTTTATGTAAAGCGTTTACAAT<br>CAGAGAAACTCGGACCCAAATCGGATAAGTGTATATTTGTGGGATATCCTAAG<br>GAAACGAAAGGATATTATTTCTACAATCCTACAGAGAACAAAGTGTTTGTTGCT<br>CGAACCGGCGTGATCCTGGAAAGAGAGTTTGTCTCTAAAGGAACCAGTGGGAG<br>GACTGTGGAACTTGAAGAAGTTCAAGAACCACAAAATATTGATCCGATGGTGG<br>AACATGAGCAGGAACCACAAGGAGTTGTGGAGCAGCCTGCTCAAGTAACACA<br>AGACCTTCGTAGGTCTGGTAGGATACGTCAAGGGCCAGAGAGATATGGATTTC<br>TCATAAGCGAGCCCGGTGACGTATTGATCATGGAGGATGATGAGCCTACAACC<br>TACGAAGAGGCCATGAAAGGGCCGGACTCTCAGAAATGGCGTGAGGCCATGA<br>GATCCGAAATGGATTCCATGTATACCAATAAAGTATGGACTTTAGTGGAACCA<br>CCTAATGGGGTAAAACCCATAGGGTGCAAGTGGGTCTTCAGGAAGACCGACAT<br>GGATGGCAATGAAATTGCCTTTAAGGGGCGATTGGTGGCAAAAAGGTTTCAGG<br>CAGGTTCATAGTATTGACTATGACGAAACTTTTTCCCCAGTCGCAATGCTTAAG<br>TCCATTCGCATCTTGCTTGCAGTAGCAGCTTATCAAGATCTATAGAGATAGATC<br>AAGGAAGTTGCTTGGCCTAAGTCAAAGTACATATATTGACAAAGTGCTGAAAC<br>GGTTCAGCATGGAAGGGTCGAAGAAGGGGTTCTTGCCCATGTCACATGGAGTT<br>AGTCTTTCGAAGACTGGCTGTCCATCCAATAAGATTGAGATGGACCGTATGAGT |

AAGATCCCATATGCTTCGGCTATTGGATCTATCATGTACGCCATGTTATGTACTA
GACCGGATGTGTCATACGCCTTGAGTATGACAAGCAGATACCAGTCCAATCCA
AGTGATAGTCACTGGATTACCGATTAAGAATATCCTTGAGTACTTGAGAAGCAC
TAAGGATGAATTCTTGATAAACGGTGGTGAGGAAGAACTCAGTGTAAAGGGTT
ACACTGACGCGAGCTTCCAAACCGATAAGGATGACTTCCGTTCGCAATCGGGG
TTTGTGTTTTGCCTGAATGGAGGAGCTGTGAGCTGGAAGAGTTCTAAGCAAGAG
ACAGTTGTCGATTCTACGACAGAGGCTGAGTACATTGCTGCATAGGATGCAGC
AAAGGAAGCTGTTTGGATCAAGAAGTTCATCACTGAACTTGGTGTGGTTCCTAG
CATAGTGGATCCCATCGCGTTGTATTGTGACAACACTGGGGCGATCGCACAAG
CTAAGGAACCCAGGTCACACCAGAGATCCAAACACATACTCAGGAGATTCCAT
CTCATTCGAGAGATAGTCGATAGAGGAGATGTGAAGATATACAGGATACCAAC
TGAGGAGAATCTTTCAGATTCATTTCTGACCCATGCACAGCGAAAGCGTGAGG
GCCACACTAGATCTATGGGCATGCGAGATGCACCAGATTGACTCTAGTACAAG
TGGGAGATTGTTGGTTAGATGCCCTAGAGGCAATCTAGTTTAGTGTGTATATCG
TTTGGCTAAACTATTCATTATGATTGGATATTTGGATATCAATATAATGAAGTCC
TTAGATTACAAATGTTGTTATCTTAAATTATCCCTGGTCAAGTATAATGATTAAT
AGGACGATCACTATGCATTGAGACTAGTATGTGGGTAGGTTGATATGGAGATAT
CAACGCTAACACATGGGTATGCATAAGGAGTTATGGCATACTGGAGACCCACT
TATGAGAAGGCTACATGCAAGCTATGTCAAGTCACAAGCGCTTCTCATAGTGA
CAATGGTGTTGTGGTCCTCAGACTTGAAGTCACTAGATACTCTAGTTGTGAATT
GTCATGCTTTGATATCGTCAAACGTTAGTCCGTAACTGGTTAACTATAAAGGCG
ATCATTGGGCATGACATGAAGCAAGCTGAGAGATGGTAGTGATGTAGAGAGG
ATTTGTCCCTCCCACATAAGCGGGAGTTAATATCCGAGGCCACTCGATGAGTAA
GACTTAAGGAGTGTCTGGCCAGACCCAGATAAATCAATAGAGATTGATGTTAT
CTGCTCGGTTAAGTCTACTCACAGATCGAGAAACTATGGATTGGATATACGAGT
GTGACATCGTGTCTCGTGTCCAATCCGGATATAGAGACAAAGGGATAAATACA
ACACGGTAACATTGGTCACAAGGTTTGTCTGATCACAGACTATTCCTATTACTT
GGGTAGCACTGATGGGTTGCTAGACTCCGCTCTTTGCTTGTAATATGATTACTGC
CAACGTAATAGGAACCTACAGGGTCACACACCAAGAGACTAAGACTAGAAGT
AGAGGTTAATTAGTTGGACTAATTAACATATGAGAATCAGGGACCAAAATGCA
ACTACGCGCAAACTTAGTGGGACTAAGTAGCAAGAAGGACTAAAACGAAAGT
TGACCCAAAGTGTTGGGGGCAACTAGTAGTGGGGTTGTCTAAGTAATTAATAA
ATAGTTATTAATTACTAGTGGGCTTGTTTTAATATTAATAATGGGTTATTAATTA
TTGACCGGTTTGACTGGTTTGACCAGTTTGACCGGTTTGACCAGAGTTTGACCA
GTTTGACTGGTTTGACCCAGTCTTCGAATTTTAATTAAAATTGACCAAGCGCGA
TAAAATTGATTATAATTGTTATATGTGTTTATCGCATGGTGGGTGGATGTGGCCT
TAGATGTGGATGTTTAAATTGCGTGGTTTGTTTGGTTTGGTTTACGGCCTGCGTG
TCGTTAAATTAATGTAATAATTAATTTTGTTGCGGGCTGCGTCCCATCTTCTTCCT
CGCATGTGAGGCTCTCTATTTAGTTTAGTAGTAGACTAGTATTAGATAGCGAAG
TGAAGAGAAGACCAAAGTCAAGGAAACCGCATGGCAATGAAGGACGCCACG
AGCATTGGATGCATGGACTAAAGACGGAGGCGCATTGGATGAGCTTGCTATTTT
AGGACCTTCTAGATGTTCCCAACGGCTTGGGAATATCTTAGTTTAGAGGCCACA
TCCACACCACGTTAATTTTATGTATGTATGTATGATGCATGCTTTATCTTGTTTTG
TGGTTTGGTGTATGATGATGCATGCAAGATAATAAACAAGATTAAATCTATTTT
ATTTGGATTCGCCCAAATCCATTTAAATAAGATTAGTTGATATGATCAACAAAT
AATTGATAATGATCAATTAAATAATAATTAACGACAAAATCCCTCAAACGACA
ATATTACATTTTAGTCTTCTTTTGGTAGCACTCGTTACTATCTAGATCATTTGTCA
CGTGTGCGCCACAGCGGAGCGATGACATTTAATCATGATAGAACGCGACGCCA
CAAGCTAGTAATGTGGGATTGCTATCGAAAGGTCAAATGTAACCGAGCGACCT
CAATAGGATTGAAAGCTTGGAGGCAAGGACAATTGGGTGATTATTTATGAGAT
AAATAATTAATAAGGAGTTATTCCCAATTAAACCAAGAGTTGCATTGGATGCA
ATTGGAAACGGTTCCTACCTAAATAACCAACTTCTGGGTGATCCGCTCAGCGGC
TCATGGGGAGGCGAAATATGGATCTTGGCCCACTAGAGAATATAATGATATTTT
CCGCATTAATACTTGAGGGTTATTAATCCGAGAAAAATAGTGGGAGCTATATTT
AAATAATTTAGCCATTATTTATTATAGTTTGAAATACACACTAAATGTATATTTC
TCGTTACCTTGTAGAATTAAATAGCAAACAACAAACATCGACAATATTTATGCG
ATCAGTCCTTGAGAAGGACAAACTGTCTGGAAAGGGTTTCCAAGACTGGTACA
GAAACTTGAGAATTGTTCTCGAGCAAGAAAGAAAGTTGTACGTTTAGAGCAGA
AAATTCCAGAAGCACCACCATGCCAAGGGTTGGCCACTGATCTCATCCTGCAA

| | | |
|---|---|---|
| | | ATTGTTGTCGGATAGTTATAGTCAGTTTATTCTGAACTTTAACATGAATGAGATT<br>AGTAAAAGTCTGCCAGACTTGCTCAATATGTTGAGGACCGCCGAACAGAGTAT<br>TGACTAAAGGCAAGACCATAATGATGGTCAATATAAGGGAGAAGGCAAAAGG<br>TT |
| Pol _C 37/0.36 | 4727 | GATGAATAGATTTTTTGGCTTTTGCACAGCGGGTTTCTTGCACTCTGTCGCCCTG<br>TGCCCTGGTTCACCGCACCTGTAGCACTTCATACCACTGCTCGAGGCTCCAGGT<br>ACTGCCTGTGTGGATGGACGGGGCTCCTGAGGTCGAGCCGATCGATTGGTTTGA<br>TCGGTTCTCACTGCCGGTTTCTTGCCTTGCTGTTTTTCCGCCGTGAGGGCTCTCTG<br>GTACGCTTCTGAAACTGACCAGAACGTTTGAAGGCTTAACGCATCTTGCAGAGC<br>TTGCCGCAATCCGGACAAGTACCGCGCGACTAGCTGCTCCTCTGTCTCGGACAG<br>ATCGTTACGGGCAATCAACTGGTAGAACTCCTCCGTGTAATCTTCCACGGATTT<br>AACACCTTGCTTCAACGTGTGTAGGCGTTGATAAAGGGTTTGGCTGTAGCCGAA<br>GGGAAGAAAGTGGGCTTTCATCTTCTTCTTCATCTTCTCCCAATCAGCGATCTTT<br>GGTTTTCCTTGCTTCTCCCGAGAACGACGCAACTGCTCCCACCATGCTGAAGCA<br>CGACCCTTCAGCTTGATCGCAACCAGCTTCACCTTCTCACGTTCAGAAACCTCC<br>TTGTACTCAAAAACACGATCTACCTCGTTCAGCCAATCGATGAATCCATCGGCT<br>TGCAGAGTGCCTGAATAACTGGGGATCTCCACCTTGAAGGCTAATCCTCCGCGG<br>TGATCATCGCGACCGCGATACCCCCGAACCCTAGGTTCGCGGTGGTATGGGTTA<br>TCGAACGACGATGTTGAACCGGGATCAGAACCTTCATGGTCATCCAACTCTTGC<br>GCTGCCAGGCGCCGAGTTAGGTCTGCGATCTGCTGCTGCATAACTTGGTTCTGC<br>CGCTGCAGATCTTCTATCACCATCACGTCTTCGAGGCTACGCTCTCGATGGGGG<br>GCGTCTTCTGGCAAGGCCTGCCCACGTCCACGGTTGCGTTGCTGACGTCTCATA<br>GCGGATGATGATCTTCCTCCCACGTCGAACTTGCTCTGATACCAACCTGACGCC<br>GGGTTATTCAACCAAGCGAGGGATCACAGCGGAATCAACAATGAAAAAGGGC<br>AAAGAAACTAGGGCACAAAGCTCTCTAGAATTCTGAAAATATTCAATATCAAA<br>ATCTATCAATCAATAACGTTTTACAGCCTTTAAATAGATGAAAGTACTAAACCC<br>TAATCCTAAACATAAACGGAAAAGACTAATAAGTCCTTACAAGAAACTGAAA<br>ATTGCAACTAAACCCCTGAAAACACAAAAACGCGATAATAACCCTCTAGATGA<br>AGTCAGACGGCCGAAAACCCTCGTACGTCATGGCGAAGCGATGAGTTTTGCTC<br>AGAGCACCGTTTCTGCCCAGCACACGAACTTGCGGGTTTCACGGGAATCTTCCT<br>GCATCAGTCTCCCCGGGTTGGAAAGAACTCGTCCTCGAGTTTAATCCGTCGTCA<br>TCAGAATCCATGAAACACCGTGTGAGGTGTTTGACATTAAACACGTCTGACGTC<br>TTCAGGTGACTAGGAAGCCGCAACCGATAAGCATTGTCATTGATCTTCTGCACA<br>ATCTCGCAAGGCCCAATCTTCCTGTCCTTCAGCTTGTTGTATTCTCCCACAGGAA<br>AACGATCACGAGTCAATACAGCCCATACAAAATCACCAACATCAAAAAGTAC<br>CTGACGACGATGCTTGTCGATCCGGGCCTTGTACTTGCTGTTGCTCTCCTCAATC<br>TTGCTCTTCACTTGATCTTGAATGGCCTTCAAATGATCCGTCATCTCCTCAGCCT<br>TAGGATGAATACGCCCCACACGTGGAATAGGAACCAAGTCCAATACCCCTGAC<br>GGGTTCTGACCATAGACAATCTGGAAGGGACTCATCCCCGTGGTTCTGCTCACC<br>GATCTGTTGTACGCGAACTCTGCCTGAGCAAGCGCCAAATCCCATTGCTTTGGC<br>TTCGAACCTGCTAAACATCTGAGGAGATTCCCGAGACTGCGATTCACCACCTCT<br>GTCTGTCCGTCGGTCTGCGGGTGATACGCACTGCTGAAGTTCAACTGAGTACCC<br>AATTTCTCCCAAAGGCTCTTCCAGAATTGACTCATGAACTTCGTGTCACGATCT<br>GAAGTAATGGACCGTGGCACCCCATGCAGCTTCACAATTTCTCTGAAGTAGAG<br>GTGGGCTATCCGCACTGCATCCATTGTCTTCCTGCATGGTACAAAGTGTGCCAT<br>CTTGGAAAACCTGTCAACAACAACAAGAACAGAATCTGCCGCTCTCTGTGTGC<br>GGGGTAAGCCAAGCACAAAATCCATACTAACATCAAACCAAGGCGCCTCAGG<br>GACAGGTAAAGGAGTGTAAAGACCTGCATTAGTAAGAACTCCCTTCGAACGCT<br>GACACACATAGCAACGATCCACATAGTGTGCCACATCTGAACTCAACTTTGGC<br>CAATAAAAATCAGCAGCTACAAGAGCCAATGTCTTGTCTCGACCAAAATGTCC<br>TTCCTGGTGAAGCTCTCTAATAATCTGCTGCCTCAAAGAACAATCTGGAACGCA<br>CAAACGCCGTCCCCGAAACAGATAACCATTATGCAAAACAAAATCATGGCGCT<br>GACCAGAAGTTACCTCAGCAAGGATCTTCCCGAAGGAAGGGTCTGCCTCATAC<br>ATCTCTGCAAATACCTCAAATCCAGGGACTTGGTTGCTCATGGAGGTAAGAAG<br>CATGTTACGACGACTCAAAGCATCAGCTACCTTGTTCAAGCTTCCTGCCTGGTG<br>CTTCAATGCAAAGGTGAATTCCTGCAAGTAGGCCACCCACTTGGCATGTCGCCT<br>ACTCAACTTGTGCTGCCCATTAATGTACTTCAACGCCTCATGATCAGTGAACAA<br>GACGAACTCCTTCTGCACTAAATAATGACGCCAATGCTTCAAGGTTTGAATAAT<br>GGCGTAGAACTCTAGATCATAAGTCGAGTAATTTTTCTTGGAGCTCGACAGCTT |

| | | |
|---|---|---|
| | | CTCACTGAAAAACGCAATCGGACGTCCCGACTGACTTAGAACACCTCCAATGC<br>CCACGCCTGAAGCATCACAATTAACCTCGAACATCTTGTCAAAATCAGGAAGG<br>GCTAAAACTGGTGCCTCTGTCATCTTCTTCTTAACCTGCGCAAAACTAGTCTCTG<br>CTTCATCCGTCCACTTGAAATCACGCCCTTTCAAACACTCAGTGATGGGTGCAA<br>TCAAGGTGCTGAAATTCCGGATGAATCTGCGGTAGAATGACGCTAGCCCATGG<br>AAACTGCGCACATCGTGCAGTGTCTTAGGCCTAGGCCAGTTCACAATTGCTTCT<br>ATCTTCGATGGATCTGCAAGTACACCATCAGTAGACACTATAAAACCCAAAAA<br>CTGCACAGAAGTAGTGAAAAAGAAACACTTCTTCCTGTTAATGAACAGATTCT<br>CTGCCTTCAATGCTTCAAAGACAGCTCGAAGATGATCAAAGTGGACATCCCAC<br>GTAGGACTGTAAATAAGAATGTCATCGAAATAAACGACAACAAACTTTCCCAT<br>GAAAGGCCTCAAAACCTGATGCATGAACCGCATGAATGTGCTGGGTGCGTTGG<br>AGAGACCGAAAGGCATCACCATCCACTCGTACAATCCATGTTGCGTCTTAAAC<br>GCAGTCTTCCACTCGTCTCCTTGCTTGATCCGAATCTGATGGTATCCACTCTTGA<br>GGTCAATTTTTGAGAAAACCTTAGAGCCGGACAGTTGGTCTAGCATATCGTCAA<br>GCCGTGGAATGGGAAACCTGTACTTCACGGTGATCCGATTGATGGCTCTGCTGT<br>CAACACACATGCGCCAAGAACCGTCCTTCTTGGGAACTAATAGGGCAGGTACT<br>GCACAAGGACTCATGCTCTCCTTGATGTATCCCCTCTCAAGCAACTCAACCACC<br>TGTCGTTGCAACTCTTCTGCTTCCTTAGGACTGAGACGATATGCAGGTTTGTTTG<br>GCAGACTAGAACCTGGGACTAGGTCTATCTGATGCTGAATATCTCTCAATGGTG<br>GCAAACCAGGAGGGAGGTCCTCTGGCATTAAGTATGAGAATTCATTCAAAACC<br>TGCTGCACTTCTGGGGGTAAATCAGAATCCTCACTACCTGCACTGCTATCAGAA<br>GGAATCAAAGCATAAACATACTTTTCCCGCTGAACCTCTCCCAAGAACTGGGA<br>TCGAGAAAGTAAGTTAGCTGGCGCGGTTGGGGTTGGAGTGGGACCCTCCCGTC<br>GCGGTGCTAGCACAATTTTCTTTCCCTTCATAACAAAAGAATGGGTGTTCTTATA<br>CCCATCATGAAACACACGACGATCATACTGCCATGGACGACCCAATAAAATAT<br>GACATGCATCCATCTTTACCACATCACACCAATGACTATCATGATACTTATTAC<br>CAATTGAAAAGGAAACAAGACATCGTTTATCAACTGTTACCTCAGAGCCTTTTA<br>CTAACCATGACAGTTTGTAAGGCGCAGGATGATGCTCCGTCTTCAACTGCAATT<br>TCTTGACAGCTTCTTCGGAGACTATGTTGTCGCAACTGCCGCCGTCTACGATCAT<br>CTTGCAAACCTTCTTCTCAATCGTGCAGGTGGTGTGAAAGATATTGGTTCTCAA<br>CCAATCATCTTCCGATTCGCCCTTGGGAGTTAGCAAACTCTTACGGACGACCAG<br>ATTCTCGCGACCATCTCCATAAAGCACATCTTCATCTTCATAGTCGTCATATACG<br>GGGTCTCCCTGGTACTCAACTTCTGCAATCTCGTCACTTGCACCTTCTTC |
| Pol _C 77/0.073 | 4618 | CCGCGACGTGATCGTTCGCGACGACGAAGATTCAGGAGCTAATTCCGGGTAAT<br>ATTTTAGCAATTAGTTTGCTTGTGTTCCACCGTTTGGTGAGGTTTACTTTTACCCG<br>TCTTTATTAGTTCCTAGTTTGTTTACCCACATTATTTGTGGAGGATTGTATTCCCA<br>TTATTTGTTATAGTGGAAGTTCTTGTGGGTTTTTTTGTCCCTCTCGTTAAGAGGGT<br>TTTTCCCACGTTAAATTCTTGTGTCCTACTCTTCTATTTTTATTCCGCACTTGTTCT<br>TGTCGGTAGGATTCGCATCCGGGTTGTTGTTTCCTCCCCGTAACCCAACAAAAG<br>TGGTATCAGAGCCGAGGTTCAATGGGGGAATCAACGGGTTCCATGATCAACTT<br>GACTGCTACAAACTACTTGATCTGGAAGCCTAGGATGCAGGACATGCTTTATTG<br>CAAAGATTTGATGGACCCCATTATTGCCAAAGGGGCCATGCCAACTGACAAGA<br>AGAACGATGAGTGGACCAAGATGAACATGAAGACAGTTGGTCATATCCGGCA<br>GTGGATTGACAACTCGGTGTTCCACCATGTTGCTCAAGAGACGGATGCCTACCA<br>ATTGTGGACCAAGTTGGAGGCCATGTATGAGAGGAAGACTGCCAGGAACAAA<br>GCGTTGCTTATGAGATCGCTTGTGAACCTGAAGTACAGAGACGGACAACAGAT<br>TGCTGAGCACTTGAGTGAGTTCCAGAGTCTCATCAACCAATTGGCTACCGTCAA<br>GCTCAATTGGGATGATGAGACACATGCCTTGTTGCTGCTTAGCTCCCTACCGGC<br>CTCGTGGGAGACGTTGGTCGTGTCATTGAGCAATTCTGCCCCCGAAGGAAAGCT<br>GTCCATGAGTATGGTGAAGGATGCGCTGTTTAATGAGGAGGCAAGGAGGAAAG<br>AGATGGGCTCTGACAATTCGCATGCTCTTGTCACGGAGAATAAAGACAAGAAC<br>AAGAATAGAGGGAGAAGCCAAACAAGGAACAACGACAACAACAACAGAGAC<br>CGCAGCAAGTCTCAGCCAAGATCCAAGATCAAGTGCTACAACTGTGGCAAGAA<br>GGGGCACATGCAGAGAACGTGCCGATTTCCAAAGAAGGATCAGAACCACGGC<br>GAGAAGCGAAAGAACCAGAAAAATGACAATAGTGAAGATGATGATACAACTG<br>CCATAATCTCAGAGGACATTGTGGTACTCTCTGTGGGAGAAGAAGAATGCCTTC<br>ACGTTGCAGCAAATCAAGAGACTGAGTGGGTTGTTGATACAGCCGCTTCATAC<br>CATGCAACTTCGAGACGAGATTTCTTCACTAACTACAAAGCTGGTGACTTTGGT<br>ACTGTGAAGATGGGTAATACGAGTACCTCGAAGATCGCAGGGATTGGAGATAT |

| | |
|---|---|
| | TCGCATCCAAACCAATGTTGGATGCACGATGGTACTGAAAGAGGTACGTCATA<br>TTCCTGATTTGCGGCTGAACTTGATTTCTGGTATCGCTCTGGATCGCATGGGTTA<br>TGAGAACTACTTCGGTAGTGGGAAGTGGAAGCTGACCAAGGGATCGATGATTG<br>TGGCTAGAGGACAAGCTTGTTGCACGCTCTACAAGACTCATGTGAATGTACTTG<br>GTGATAGCGTGAATGCAGTTGAGGATGCAGCATCACCAAATTTGTGGCACAGG<br>AGGCTTGCTCACATGAGTGAGAAAGGATTGAAGATTCTGGCAAAGAAGTCTCT<br>AATTCCCTTTGCCAAAGGTACACCACTAAACCCTTGTGATTATTGCTTGTTTGGT<br>AAACATCACAGGATTACTTTTAGTTCCTCTGCTAAACGAAAAACTAAGATACTG<br>GAGCTAGTGCACTCTGATGTGTGTGGTCCCATAGAGGTGGAGTCTATTGGTGGT<br>TGCAGATATTTTGTAACCTTTATTGACGATGCTTCACGTAAAGTGTGGGTGTATT<br>TTCTGAAGACTAAGGATGAAGTATTTGATTACTTCAAGAAGTTCCATGCCATGG<br>TTGAAAGAGAGACAGGCAAACCTTTGAAGCGTCTGCGTACAGACAATGGTGGT<br>GAGTATACTTCAAAGGAATTTGAAGCCTATTGTGATGCCCACGGTATTCGGCAT<br>GAGATGACGGTTCCAGGTACACCACAACACAACGGCGTAGCCGAGAGGATGA<br>ATCGGACTATTGTAGAGAGAGTTCGATGTATGCTCAGAATGGCAAGGCTGCCA<br>AAGCCATTCTGGGGTGAAGCAGTTCGTACTGCCTGTTATGTGATTAACAGGTCT<br>CCATCTTCTCCTTTGAGTTTTGAGATTCCAGATAAGATATGGTATGGCAAGGAC<br>GTATCATACTCTCATCTGAAGGTATTCGGCTGCAAGGCATTCGCGCATATACTC<br>AAGGAGAAACGACTAAAGCTTGATGATAAAGCCAATCCGTGTGTTTTCGTGGG<br>CTATGGAGATGCGGAGTTTGGTTACAGATTATGGGATCCAGTGAACAGGAAGC<br>TGATAAGAAGCAGAGATGTGGTTTTCTACGAGAGCCAGACTATTGAAGATATT<br>GAGAAGTCTTCAAAGTCAAACGTAGCAGGTGCTCCCGACTTCGTACCAGATAC<br>CTCACCTAGTGCCATAGGTGGAGAAGAGATACCTGGAGAAGCTCCAGAAGTAG<br>AAGTTCCAGAGATGGAGGATGATGATGATGATTCCGATACAGAGAGTGTTGAG<br>CAGGGGGAGCAGCCCCATCCACCAGAGGATGATGGACCTCAGCCAAGAAGGT<br>CCACGAGGAATCCGCAACCATCAGCCAGGTACCCATCGTCTGATTATATTTGG<br>TTACTGATGAGGGGGAGCCAGAATGTTTCCAGGAGGCGCAAACTCATAAAGAT<br>AAATCCAACTGGATGAAGGCTATGCAAGAAGAGATGAGTTCCTTGCATAAGAA<br>TGATACTTATGAGTTAGTGAAGCTCCCGAAGGGAAGAAAAGCACTAAAGAAC<br>AAGTGGGTCTTCAAGCTAAAGAAGGACAATGATGGGAAACTAGTGAAGTACA<br>AAGCTCGATTAGTGGTCAAGGGTTTCGGCCAAAAGAAAGGTATTGACTTTGAT<br>GAGATCTTTTCGCCAGTTGTTAAGATGACTTCTATTCGAGTAGCCTTAGGGCTAG<br>TAGCTAGTATGGACCTCGAGCTTGAACAACTTGATGTGAAGACTGCCTTTCTTC<br>ATGGAGACTTGGAGGAAGAGATTTACATGGAGCAGCCAGAGGGATTTGAAGT<br>GAAAGGGAAGGAGAACATGGTTTGCAAGTTGAAGAAGAGTCTATATGGCCTTA<br>AGCAAGCGCCGAGACAGTGGTACAAGAAGTTCGACTCATTCATGGTGAGTCAT<br>GGGTACAAGAGAACACAAGCAGACCAGTGTGTTTACATACGAAGATTCCCCGA<br>TGGTAACTTCATCCTATTACTACTATATGTCGATGACATGTTGGTTATCGGTAAG<br>AATTCGGAGATGATTGGCAAGCTGAAGAAGGAGTTATTCAAGTCTTTCGACAT<br>GAAAGACTTAGGTTCCGCTCAACAGATTTTGGGCATGAAGATTGTTCGTGACAG<br>GAAAGCAGGGAAGCTTTGGTTGTCGCAAGAGAAATATGTTGAACGCGTGATCG<br>ACAGGTTCAACATGAATAGTGCTAAGTCTACTAGCACTCCACTTGCCAGTCACT<br>TCAAGTTGAGTAAGAGATCGTGTCCCTCTAGCAAAGAAGCTAGAGATAAGATG<br>GCAGCTATTCCCTACTCCTCTGCAGTAGGAAGTTTGATGTATGCTATGGTTTGCA<br>CCCGACCAGATATTGCTCATGCCGTTGGCGTAGTGAGCAGGTTTCTCTCTGACC<br>CTGGGAGAGAACATTGGGAAGCAGTCAAGTGGATTCTGAGATATTTGAAGGGG<br>ACTTCCAAATGGTGCTTGTGCTTTGGTGGTTCCAAACCAGTCTTGGAAGGATTTA<br>CAGATGCCGATATGGCGGGCGACCTCGATTGTCGAAAGAGCACTTCAGGATAC<br>TTGTTTACTTTTGCAGGGGGAGCTGTATCGTGGCAGTCCCGACTACAAAAATGT<br>GTTGCTCTGTCAACAACCGAGGCCGAGTATATTGCTGCAGCGGAAGCTGGCAA<br>AGAGATGCTTTGGATGAAGCGGTTTCTCCAAGAATTGGGTTTGAAGCAAGACG<br>AGTATGTGGTTCATTGTGATAGTCAAAGCGCTTTGGACTTGAGCAAGAATTCCA<br>TGTATCATTCTCGCACGAAGCACATTGATGTTCGCTATCATTGGATACGCGAAG<br>TGATAGAGAAGCAGCTGATGCAACTCAAGAAGATTCACACTGATCAGAATCCG<br>TCCGACATGCTGACAAAGGTGGTGACCAGGGACAAGTTCCAGTTCTGTAGAGA<br>CAGAGCTGGGATGGACTCCAAGTGAGGAGTCATGGTGGTGTTCCCTCCTCTAGA<br>TGGGCTCGGAGGGGGAGATTTGTTGGGTCCAAGCCCAGTCCATGAGTTGTGTGA<br>GGAGGTGATGTTTTTGGCAATATTGCCTAATTGGTTTGTTTTCCACCTCCCTACA<br>CTACTATATAAGCTCATGCCTTGGAGCTTTGTAGACACACCAAAAAGAGTACCT |

| | | |
|---|---|---|
| | | CTTATCAAGTAAGAGAGTTATTTTTTGCTTTGTTCGCCGGGTTGACGACGGGCA<br>AGAGACGAAGAGTT |
| Pol _C 82/0.068 | 4390 | AGAAGAGAGATTGCCTGAAACGAGGACAACCGGAACAGACTGGATTGGTAGC<br>CGTGGTAGAGAAGAGTAACTCGTCCGAGGACGATTACGTTCTATCGGCGGATT<br>GCATCACACCCCAGCCGGATGCGTGGGTTCTTGACTCCGGAGCTTCTTATCACA<br>TCTGCTCTAACAGGGAGTTGTTTTCAAGCTATTGCCAAACGGATGGAGGTTCTG<br>TTCACATGGCGAATGGGGCTGTTTGTAAGGTGATAGGAAGCGGCTCAGTTAGAT<br>TCCAGAATCATGATGGGAAGGTATGTACTTTGAAGGAGGTTAGACACGTTCCA<br>CTAGTCACGAAGAATTTGATCTCCCTAGGGCTACTCGACAGCAAGGGTTTCAGC<br>TTCAAAGGTGAAGGTGGAGACTTACACGTCTACAAAGGTTCAAAGACACTACT<br>CAAGGGAGTTAAGGATGGGACTCTATACTTATTGAAGGGTTCGGCGATCACAG<br>GGTCAGTTGGAGTTGTCTCAGCCGAGGTACAAGAAGCTGATCATACTAAGTTGT<br>GGCATATGAGACTCGGTCATATGAGCGAACGTGGGATGCAGGAACTACACCGG<br>AAGGGCTATCTCGCCGGGCACACGCCCAAGAACCTAGGGTTTTGTGAGCACTG<br>CGTGTACGGGAAGCTACAGCGTAGCAAGTTCCCAAAGGCGGTTCACCAAACGA<br>AAGGCACTCTTGATTACATCCATTCGGATTGCTGGGGTCCAGCTCGAGTTGAGT<br>CGATTGGCGGTCACAGATATTTTCTGAGCATTATTGACGACTATTCAAGGATGA<br>CTTGGATTTTCGTGATGAAGCACAAGAGTGATGCATTCGGCAAATTCAAGGAGT<br>GGAAGACACTAGTAGAGAATCAGGCGGGAAAGAAGGTGAAGAGGCTGCGCA<br>CGGACAACGGCTTAGAGTTTTGTTCATCGGAGTTCGATGAATTCTGCAAGGATC<br>AGGGAATAGCTCGCCATCACACCGTTCGGCACACACCACAGCAGAATGGTGTA<br>GCCGAAAGAATGAATCAGACTCTTTTAGCGAAGGCAAGGTGTATGCTTTCGAA<br>CGCAGGTCTTGCTAGGCGTTATTGGGGAGAGGCAGTTTCTATGGCCTGTTATCTT<br>GTAAATCGGAGTCCTCACACTGGCATTAAGTGCAAGATACCGATGGAGGTATG<br>GTCCGGAAAAGCTGTTGATTATTCGAATCTTAGGGCTTTTGGATGTGAAGCCTA<br>TTATCATACAGATGATGGGAAGCTAGAGCCAAGAGCGAAGAAGGGGATTTTCA<br>TGGGTTTTGGGACGGGTGTGAAAGGCTACAGGATTTGGTCGGATGGGAAGATC<br>ATACTCAGCAGGAATGTGGTTTTCAATGAGAATCCGATGCTAAAACCAACCGT<br>GGAGATAGAGATTGAAGCTGCAAAGAAGAAGGTGGAGGCAAGGTCAGGTTCG<br>GCTAATCCTAAAGAACCTGAAACGCCACCATCACCGCCGCCAGAAACTGAAG<br>AGCAAGAATCGGAGGAGTCTGAAGAGGATACGCCATCAGAGGAACTAGTACA<br>TGAGAGTATTGCTACGGGTCGTGCAAAGAGAACTACAGAGTTACCAGCAAGAC<br>TCAAGAATGACTACTTGGTGGGGTATGCCTTGCAAGTAGCCGAAGAGGTGGAG<br>GATGAGCCGTCCACCTATCGGGCTGCGATTACTAGTTCTGAGTCAGCACAATGG<br>ATTGCCGCGATGGGAGAAGAGATGGAGTCTCTTAGCAAGAACTCGACATGGGA<br>GCTTGTTCCATTGCCACAAGGGAGAAAGATAGTCACTTGCAAGTGGGTTTTCAG<br>AAGAAAGGAAGGAATTACCACAGCGGAAGGCATCAAGTACAAGGCAAGAGT<br>GGTTGCAAGAGGGTTCAGTCAGAGAGAGGGAGTTGACTACAACGAGATATTCT<br>CACCGGTGGTCAGACACACTTCGATCAGGGTGCTACTAGCAATGGTGGCACAC<br>CAGGACATGGAGCTTGAGCAACTCGATGTGAAAACAGCTTTCTTACATGGAGA<br>GTTGGAAGAAGAGATATACATGAGCCAGCCGGAGGGTTTCCAAGTTCCTGGAA<br>AAGAAGACCACGTGTGCAAGTTGCAGAGGTCCTTGTACGGACTAAAGCAATCT<br>CCGAGGCAGTGGTACAAGAGGTTCGATAGCTTTATGCTGAAATTAGGCTATCA<br>GAGGTGTGAGTATGATTGCTGCGTGTATCACAAGAAGTTGCATGGTGGATCACG<br>CATTTACCTAGTTCTATATGTGGATGACATTCTGATTGCAACCCAGAATATCAA<br>GGACATTAGAGAACTGAAGAGGTCTCTCAGCACGGAATTCGAGATGAAGGATT<br>TGGGGGCTGCTCGAAAAATCCTAGGAGTTGAAATCTATAGAGATAGAAAGAAG<br>AGGAAGCTCTTCTTGTCACAGGAGGGTTACATCAAGAAGATTTTGCACAAATTT<br>GGGATGCAAACCGCCAAGTCTATAGATACTCCTATGGCGTCTAACAGCAAGCT<br>AGGAATGTACACCGTTCAGACCGAGGAGGAGAAGGAGTACATGTCCAAGGTT<br>CCATATGCTAGCGCAGTTGGAAGCCTCATGTATGCTATGGTCTGCACTCGACCA<br>GACATAGCATTCGCTGTTAGTGTGGTGAGTCGATTTATGGTTCAACCGAGCAGG<br>GATCATTGGCAGGCCGTGAAGCGGATATTCAGGTACCTTAAGGGTACGTCAGA<br>CATTGGCCTATGCTATGGGGCTAATGAAGAGATCTTGGTTGCTGGGTTCTCAGA<br>TTCAGACTATGCAGGTGATGTTGATAGCAGGAGATCGATGACAGGATACGTGT<br>TCACTCTGGGAGGTTCTGTTGTGAGCTGGAAGGCGACGCTACAGCCGATGGTG<br>ACCTTGTCAACCACGGAAGCGGAGTTAATGGCATTAACGGAAGCAGCAAAGG |

| | |
|---|---|
| | AGGGAATATGGTTGAAGAATATGGTCAACGATCTAGGCCTTCATCAAGATCAA<br>GCGATAGTTTACTGTGATAGCTTGAGTGCGATTTGCTTGACTAAAGATCAGGTC<br>CACCATGAGCGGACAAAGCACATCGACGTCAGGTATCATTTTCTGAGAAACGA<br>GAGTAGAATCAAGGTGAAGAAGGTGGGGACAGCTGACAACCCCGCGGATATG<br>TTCACGAAGCCGGTTCCGGAGAGCAAGTTCAAGCATTGCTTGGATTTGCTCAAT<br>ATCATGAACCATTAGGCCCGTTGGGGCTGTCGGACCCGGTGGGGGTCCAGTGA<br>GTCTCTGTGACGAGAGACATCGGGTGCGGAGAAGGCAAGCTTTGTTTACGAGA<br>AGAGAAGCATTCGTTTCAGACTAGAGAATTCAAGTCAAGGTGGAGATTTGTTA<br>GTCGGTGTGACTTGAATTGAAAGTCAAAGTCGGTTAGGCTAGGAAACAAGTAA<br>GGGCGGCCACAATATGTTTCCAAATCAGAGTTGGAAACGTGTGGTGGTGGGTC<br>TCTCTTAGCAGTATAAATAGGACCTCTATGAGGTCAGTAGATGTATGCACAACC<br>AATTGTATTTGGCTTCTCTTGTGAAATAAAGGTCGTTCTTCGCCGTGGACGTAGT<br>TAGCACGAATTGTGCTAGCGAACCACGTAAAACTTGTGTGTTTTGTCTTTCCGGT<br>ATCGTTGTGAGGTTGAGCGTTCATAACAAACTGGTATCAGAGCTTAGGGTTCGT<br>GGTTGCTCCACCAAGAACAATGTCCGGATCTATTATGAAGATCGAGAAATTCA<br>CCGGAGGGAATAGCTTCAGTCTGTGGAAGATCAAGATGGAGGCCTTGCTCAAA<br>TCGCAAGGCATATGGGCACCTTTGACCGCGGACAAAGGAGGCATGGATGCTGC<br>ATCAGCGGAGTACAAACTGATGGAGGAGAGGGCACACGCAAACATCGTGTTG<br>TGTTTGTCAGACGACGTGATCACGGAGGTTGCAGGGGAAACAAAGGCCGCCGC<br>CTTGTGGCTCAGGTTAGAAAGTCTCTACATGACGAAGAGTCTTACTAGCAAGCT<br>GATGCTAAAGCAACGGTTGTTCACCCTACGGATGACGGAAGGTACGACTCTCA<br>AGGATCACCTTGACCAATTGAACACGATTCTGCTGAATCTGCGCAATATCGAA<br>GAAAAGGTGAATGATGAGGATGCTGCACTTCTTCTCTTGATCTCGTTGCCACCA<br>TCATACGAGAACTTCGTACAGTCCTTTATTGTTAATAAGGACAGTATATCTCTA<br>GAAGAGGTGCGGTCAGCTCTGCACGCTAGAGAGATGCGCCACAAGGGCACAG<br>GCACGAGCGCGGAGGACCATGCTTCCGGATTGATGGCGACAAAGGGAAGCAA<br>AGGTAAGAAGAACAAGAAGAAATTCTCGGGCTCTAAATTACCTAAGGATGTCT<br>GCGCTTGGTGCAAGGAAAAGGGGCACT |

**Supplementary Table S2.** List of the oligonucleotide FISH probes.

| Oligo FISH probe name | | Oligo FISH probe sequence |
|---|---|---|
| Pol_C 33 | Pol_C 33_1 | CTCATTTGCATTCCATAGGCATTGTATGATCTAAGGGATTCACTCCTA |
| | Pol_C 33_2 | AAATGTGTTTCCATCTAATTTGGTGGCCAACATGTTGGTGA |
| Pol_C 46 | Pol_C 46 | TGGGGATTAGTGTATCCCCAAAATATGACAAGT GGAATAGTTT ATCCCGCCTCTTTAATTT |
| Pol_C 67 | Pol_C 67 | GTCCAGGTTAGTGGTCATCTAGTGTTAGGAACCTACAGTTGACCTAAT TG |
| Pol_C 70 | Pol_C 70 | GGTATGAAATAAATGAACAT AAATATAAGC CATTCGTGCG TTTCGTAGTCCCACACTACCCTTTCTTATG |
| Pol_C 125 | Pol_C 125 | GTAAGTTCACATATCTCACTACAAGCTAAGAGAGAACTCCATGACTA TTAACGTCCAAATACAACTACCACTACTACATTTCA |
| Pol_C 134 | Pol_C 134_1 | GAGACTTCCATGTGCAAGTTGACACGGGGAGTGATCTACTCTGGGTT AATAGTGTTGCAT |
| Pol_C 1 | Pol_C 1_1 | CACAAATTCTAAACAGATATCACCTTTTTCTACATGATCCCTGATGAA ATGATGTCTAAC |

| | | |
|---|---|---|
| | Pol_C 1_2 | GAGACTCCAGATTGTTTACAATGTCAGTAAACCTCTTGAACATGTCTTTGATGGATTCTT |
